# Structural and Stability Analysis of GRP Family Allergens Pru p 7 and Cry j 7, Which Cause Pollen and Food Allergy Syndrome

**DOI:** 10.3390/biom15020232

**Published:** 2025-02-06

**Authors:** Jingkang Zheng, Hiroyuki Kumeta, Yasuhiro Kumaki, Tomona Iizuka, Ichiho Yoshikawa, Ami Hanaoka, Tomoyasu Aizawa

**Affiliations:** 1 Laboratory of Protein Science, Graduate School of Life Science, Hokkaido University, Sapporo 060-0810, Hokkaido, Japan; 2 Faculty of Advanced Life Science, Hokkaido University, Sapporo 060-0810, Hokkaido, Japan

**Keywords:** PFAS, GRP allergens, purification, NMR analysis, structural stability, proteolytic digestion

## Abstract

Cry j 7 is a 7 kDa cysteine-rich gibberellin regulatory protein (GRP) with six disulfide bonds. It was isolated from Japanese cedar as the pollen allergen in this study. It exhibits cross-reactivity with food allergens such as Pru p 7 from peach and causes pollen-food allergy syndrome (PFAS). In this work, recombinant Cry j 7 and Pru p 7 were successfully overexpressed using *Pichia pastoris* in a high-cell-density fermentation culture, and pure proteins were purified by reverse-phase HPLC. The characterization of Cry j 7 and Pru p 7 were performed by MS, CD, and ^1^H-NMR experiments to confirm the correct native conformation of Cry j 7 as well as Pru p 7. When compared, the results showed that Cry j 7 exhibits excellent stability in disulfide linkages and preserves its original structure up to 90 °C in various pH buffers in comparison to Pru p 7. Notably, NMR analyses indicated the greater mobility in the α-helix and loop regions of S38-C47 in Pru p 7 compared to those of Cry j 7. Furthermore, our results showed that the sensitivity of Cry j 7 to enzyme digestion differed from that of Pru p 7: Cry j 7 was more susceptible to proteolysis, while Pru p 7 displayed better resistance in the gastrointestinal tract. These variations in structural stability and sensitivity to proteolysis provide valuable insights into the allergenicity within the GRP family.

## 1. Introduction

Trees of the Cupressaceae family are distributed on all continents except Antarctica, and 162 species have been identified so far in this family [1]. The pollen from several trees is known to be highly sensitized and is associated with significant allergic diseases [2]. For example, more than 30% of the population in the Mediterranean area is hypersensitive to pollen from European cypress (*Cupressus sempervirens*) [3]. In Japan, the most common species is Japanese cedar (*Cryptomeria japonica*), and an epidemiological investigation revealed that the prevalence of cedar pollinosis has surged from 10% to 40% in the last 40 years [4,5,6]. It is well known that Cupressaceae pollen allergens are composed of several allergen groups, some of which may be involved in cross-reactivity with allergens contained in fruits causing pollen food allergy syndrome (PFAS) [7].

PFAS is a prevalent immunoglobulin E (IgE)-mediated allergic condition that occurs through a cross-reaction between pollens and foods [8]. The pathogenesis of PFAS was observed to occur in patients who inhale pollen allergens via the respiratory tract and are subsequently exposed to raw plant-derived foods (class 2 food allergy [9]) that share identical or similar epitopes to pollen proteins, corresponding to the pollen-specific IgE antibody [10]. Cross-reactions to apple fruit-derived Mal d 1, peach fruit-derived Pru p 1, etc., due to sensitization to Bet v 1 contained in birch pollen, are typical examples of this PFAS caused by the RP-10 family [11]. Besides the main pathogenic protein types, RP-10 or LTP allergens, the recently described gibberellin-regulated proteins (GRPs), which are widely distributed among all vascular plant species, including seed plants, and essential for plant defense as well as phytohormonal responses [12,13], have also been discovered as allergens involved in PFAS [14,15].

Cry j 7 is a pollen allergen from Japanese cedar, belonging to the GRPs, also known as the Snakin/Gibberellic acid-stimulated Arabidopsis (GASA) protein family [16]. It exhibits antimicrobial properties, and its amino acid sequence contains 12 cysteine residues that form six disulfide bridges in conserved positions and confers thermostability and proteolytic resistance to the protein [17]. Pru p 7, another GRP family protein, was the first GRP to be identified as a food allergen and isolated from peach fruit [18]. To date, allergenic GRPs found in other plant foods have been described, namely Pru m 7 from Japanese apricot [19], Cit s 7 from orange [20], Pun g 7 from pomegranate [21], and Cap a 7 from bell pepper [22], while subsequent extensive investigations have confirmed cross-reactivity with pollen GRPs reported in the Cupressaceae family, such as Cup s 7 from Europe cypress (*Cupressus sempervirens*) and Jun a 7 from Mountain cedar (*Juniperus ashei*) [6,16,23]. Despite coming from distinct plant taxonomic orders, there is a significant degree of sequence conservation between the primary structure of GRPs in plant food and Cupressaceae pollen, according to a comparative analysis [24].

The actual prevalence of food allergies linked to the GRP family has been less widely studied, and studies on reactions to pollen GRP are also rare. In Japan, the incidence of food allergy mediated by GRPs was 65 in 100 patients with responses to peach, and approximately 20% of patients were sensitized to Pru p 7 from Japanese cedar [25]. Another study on Cry j pollen and fruit GPR sensitization in Japan revealed that 46% patients were sensitized to Cry j 7 [26]. The connection between Cry j 7 sensitization and Pru p 7 sensitization appears to be related to severe fruit allergies [12]. Further experiments are necessary to explore the effect of GRP allergens in Japanese cedar pollinosis [24].

In this study, we applied the *Pichia pastoris* over-expression system to obtain active Cry j 7 and Pru p 7, which can be used in NMR studies. We performed a three-dimensional structure analysis of Cry j 7 and Pru p 7, and predicted the flexibility regions to obtain new insights into the structure of the GRP family. We further analyzed the effects of pH, thermal denaturation, and proteolytic enzymes on the structural stability of the GRP allergens. This information is critical for studying GRP allergens and facilitates investigations of the complex relationship between stability and allergenicity. Comparing the characteristics of pollen allergen Cry j 7 and food allergen Pru p 7 will help us understand the allergenicity mechanisms of PFAS.

## 2. Materials and Methods

### 2.1. Protein Expression and Purification

The pPIC9-Cry j 7 and pPIC9-Pru p 7 recombinant plasmid vectors were introduced in our experiments within the methylotrophic yeast *Pichia pastoris* expression system, which has the extracellular secretion pathway [27,28]. The DNA fragments encoding Cry j 7 and Pru p 7 were amplified by inverse PCR and the gene was simultaneously introduced into the pPIC9 vectors (Figure S1). The ligated pPIC9-Cry j 7 and pPIC9-Pru p 7 recombinant plasmids were introduced into *E. coil* DH5α, and the presence of the gene inserts was determined by colony PCR and DNA sequencing (3100-Avant Genetic Analyzer; Applied Biosystems/Hitachi, Tokyo, Japan). The recombinant construction was linearized with *Sal* I enzyme (Thermo Fisher, Waltham, MA, USA) and expressed into the competent *P. pastoris* GS115 strain (Thermo Fisher, Waltham, MA, USA) according to the instructions provided in the *P. pastoris* expression kit v.3.0 manual (Thermo Fisher, Waltham, MA, USA) [29].

High-cell-density fermentation of *P. pastoris* was carried out in a 5.0 L jar fermenter (MBF-500; Tokyo Rikakikai, Tokyo, Japan), following the established procedure [30]. The GS115 cells transformed with the pPIC9-Cry j 7 and pPIC9-Pru p 7 recombinant plasmids were grown in different types of fermentation media (details are given in the Supplementary Materials) at 30 °C after being initially incubated in a YPD medium. After 42 h of methanol induction, the culture was halted [31]. The expression levels of Cry j 7 and Pru p 7 were evaluated by tricine-sodium dodecyl sulfate-polyacrylamide gel electrophoresis (tricine-SDS-PAGE) followed by Coomassie brilliant blue staining.

The cells from the fermentation culture were harvested and supernatants were collected through centrifugation at 6000 rpm and 4 °C for 20 min. The pH and conductivity of supernatants were adjusted to 6.0 and 4.9 mS/cm, respectively, using 1 M NaOH and Milli-Q water. The filtered products were loaded onto a 5 mL pre-packed HiTrap SP FF cation exchange column (Cytiva, Marlborough, MA, USA) equilibrated with buffer (50 mM potassium phosphate, pH 6.0). Then, the proteins were eluted with a linear gradient of 15–36% elution buffer (50 mM potassium phosphate and 1 M NaCl, pH 6.0). Solutions were filtration (Millex-HV 0.22 μm, Merck, Rahway, NJ, USA) and mixed with trifluoroacetic acid (TFA, Nacalai Tesque Inc., Kyoto, Japan) to achieve a pH of 2.0–3.0 for reverse-phase HPLC (RP-HPLC). Proteins were purified using a Cosmosil 5C18AR-300 column (Nacalai Tesque Inc., Kyoto, Japan). Elution was performed by using a linear gradient of 15–25% acetonitrile with 0.1% TFA. The contents of each fraction were assessed using 15% tricine-SDS-PAGE without the reducing reagent. The yields of Cry j 7 and Pru p 7 were estimated by measuring the absorbance at 280 nm. The purity and molecular weight of Cry j 7 and Pru p 7 were verified by MALDI-TOF MS (UltrafleXtreme; USA). In addition, we also prepared natural Pru p 7 samples as a control; the specific information can be found in the Supplementary Materials.

### 2.2. NMR Analysis

The ^15^N-isotope-labeled samples used for structure determination were expressed within the *P. pastoris* system [32] and a medium containing 10 g ^15^NH_4_Cl [33]. Details of the isotope-labeled cultivation process are provided in the Supplementary Materials. The ^15^N-isotope-labeled Cry j 7 and Pru p 7 were dissolved in a 10% D_2_O/90% H_2_O mixture at a final concentration of 0.8 mM at pH 3.0. NMR experiments were carried out at 25 °C on Bruker 800 MHz (AVANCE NEO; Bruker, Billerica, MA, USA) and 600 MHz (AVANCE III HD; Bruker, Billerica, MA, USA). The main chain assignments of ^1^H-, ^13^C- and ^15^N-resonace were performed using the following spectra: [^1^H-^1^H] TOCSY and [^1^H-^1^H] NOESY with ^15^N-decoupling, [^1^H-^15^N] HSQC, [^1^H-^13^C] HSQC, HNCO, HNCA, ^15^N-TOCSY and ^15^N-NOESY. All chemical shift values were referenced against DSS and the following frequency ratios: (^15^N/^1^H) = 0.101329118, (^13^C/^1^H) = 0.251449519 [34]. Moreover, heteronuclear [^1^H-^15^N] NOESY-HSQC with a mixing time of 100 ms for ^1^H-^1^H NOESY provided inter-proton distance restraints for structural analysis. The experimental information is shown in Table S1. The 2D and 3D NMR spectra were processed using NMRPipe [35], and the data were analyzed using the Sparky program (http://www.cgl.ucsf.edu/home/sparky/, access on 30 May 2008).

Nuclear overhauser effect (NOE) distance constraints were automatically assigned and computed in 7 cycles of the ‘noeassign’ macro of the CYANA 2.1 software package [36]. To rectify the automatic NOE signal assignment, we extracted restraints between the medium- and long-range residues and used a pseudo-atom for all-optical isomeric protons. As a result, the distance restraints had a fixed upper limit value of 6 Å. The final ensemble of 20 structures was chosen based on CYANA target function values after the 30,000 simulated annealing steps that were used to generate 100 structures in each “noeassign” cycle [37].

### 2.3. Circular Dichroism Spectroscopy

All CD measurements were performed via a Jasco J-725 spectropolarimeter (Jasc.o, Japan) using cuvettes with a 1 mm path length. Freshly made 0.1 mg/mL (14.63 μM) Cry j 7 and 0.1 mg/mL (14.47 μM) Pru p 7 proteins were dissolved in 50 mM PBS at pH 7.4. Spectra were acquired at a 50 nm/min scan rate with 4 scans taken from 260 to 190 nm registered and averaged at room temperature, 25 °C.

For pH and thermal denaturation treatments, pure Cry j 7 and Pru p 7 powders were diluted to a final concentration of 5 mM in a 200 mM Na-citrate (pH 3.0) stock solution. At pH 6.0, 8.0, and 10.0, Cry j 7 and Pru p 7 samples were directly dialyzed against the respective buffers (pH 6.0 and 8.0: 50 mM Na-phosphate buffer, pH 10.0: 50 mM Na-carbonate buffer). The temperature of samples at 10 μM was increased progressively at a constant rate of 2 °C/min from 20 °C to 90 °C and subsequently cooled back down to 20 °C. Signals at a wavelength of 210 nm were monitored with a step resolution of 0.2 °C [38]. The mean residue ellipticity values, *θ*, were calculated using the formula below [39]:

$$\theta =\frac{\theta \mathrm{observed}}{10\times n\times C\times l}$$

where *n* is the number of amino acid residues, *C* is the peptide concentration, and *l* is the optical pass length of the cell.

### 2.4. Proteolytic Degradation Assay

GRPs at a 0.5 mg/mL final concentration were individually incubated with 0.1 mg/mL thermolysin at 30 °C in a solution containing 20 mM HEPES, 200 mM NaCl, and 5 mM CaCl_2_, at pH 7.4 [40]. Reaction aliquots from the proteolytic degradation assay were taken out at different intervals, and then proteolysis was quenched by the addition of 12.5 mM EDTA. The aliquot samples of GRPs were compared to those under tricine SDS-PAGE, and gel bands were quantified using Image J software 1.45 (http://rsbweb.nih.gov/ij/, access on 29 October 2011) [41].

Alternatively, the proteolytic stability against endo-lysosomal proteases was assessed by employing pure Cathepsin S enzyme (human, recombinant from *E. coil*, Sigma. Aldrich, St. Louis, MO, USA) [42]. Proteins were incubated with Cathepsin S in a digestion buffer (0.1 M sodium acetate, pH 5.0, 0.1 M NaCl, 5 mM EDTA, and 2 mM DTT) at 37 °C for various durations. At each time point, the reaction was halted by boiling for 5 min at 95 °C [43]. The degradation characteristics were analyzed by tricine SDS-PAGE and MALDI-TOF MS.

### 2.5. Stability to the Simulated Gastrointestinal Digestion

In vitro gastrointestinal digestion was carried out as previously described [44]. Briefly, GRP proteins were subjected to pepsin (Sigma. Aldrich, St. Louis, MO, USA) digestion in a 1:20 *w*/*w* enzyme-to-substrate ratio in 0.15 M NaCl, the pH of which was adjusted to pH 2.0 with 1 M HCl. The incubation lasted for 2 h at 37 °C and it was stopped by raising the pH to 7.4 by adding PBS [45]. For intestinal digestion, the product mixture resulting from the gastric digestion reaction was further subjected to trypsin (Porcine, Sigma. Aldrich, St. Louis, MO, USA) and α-chymotrypsin (Sigma. Aldrich, St. Louis, MO, USA) digestion in an enzyme-to-protein ratio of 1:50 (*w*/*w*) in 0.05 M potassium phosphate buffer, the pH of which was adjusted to 6.8. After 48 h of reaction at 37 °C, the process was terminated by heating the mixture to 95 °C for 2 min [46]. The degree of gastrointestinal digestion was monitored through tricine SDS-PAGE and RP-HPLC.

### 2.6. Epitope Prediction

To computationally predict the epitopes of Cry j 7 and Pru p 7, we primarily employed epitope prediction methods from the immune Epitope Database (IEDB) [47]. We utilized IEDB MHC class II binding sites and the online tool ProPred [48] to predict Th-cell epitopes. To acquire the high-scoring peptides, we set a 5% threshold for ProPred. Our analyses focused on common human alleles (DRB1*0101, DRB1*0301, DRB1*0401, DRB1*0701, DRB1*0801, DRB1*1101, DRB1*1301, DRB1*1501 and DRB5*0101), which are alleles considered representative in humans. Fewer than 15 residues were evaluated for T-cell epitopes. Simultaneously, the latest version of the BepiPred 3.0 website was considered for B-cell linear epitope prediction, with the threshold set to 0.15 [49].

Additionally, the Disco Tope 2.0 dataset is a method for predicting discontinuous epitopes from 3D structures of proteins in PDB format [50]. By uploading the PDB file of the NMR structure to the website (http://tools.iedb.org/discotope/, access on 27 December 2012), the PyMOL 4.6 software displayed the corresponding epitope regions.

## 3. Results

### 3.1. Expression and Purification of GRP Proteins

**Figure 1:**
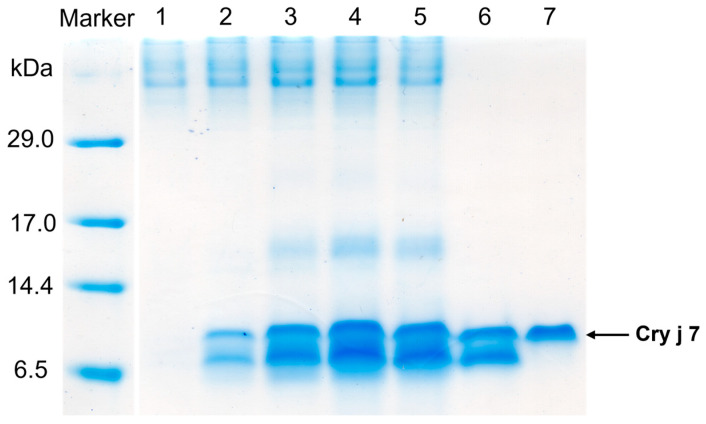
Tricine SDS PAGE analysis of Cry j 7 in fermentation supernatants from *P. pastoris* and purification steps. Lane marker: protein molecular weight mark. Lanes 1–5: a total of 20 μL of supernatant samples taken at 0, 12, 24, 36, and 42 h of induction, respectively. Lane 6: the fraction eluted at 28.0–29.0 min via cation exchange chromatography (CIEX). Lane 7: the peak fraction (17.5–18.0 min) obtained from RP-HPLC.

To achieve high yields, we performed high-cell-density fermentation in a jar fermenter, beginning with basal salt medium and involving medium supplementation with ammonia and glycerol. The total methanol induction period spanned 42 h. Following culture, the yeast cell density of Cry j 7 and Pru p 7 increased to 220 g/L and 290 g/L, respectively. A 7 kDa protein band was identified by tricine SDS-PAGE analysis of the culture supernatant (Figure 1). Two bands with similar molecular weights were observed, but upon reduction, these bands merged into one, suggesting that they are probably different molecular species of the same protein with different disulfide bridge contents. We also observed that protein expression started gradually 12 h after methanol induction and reached its peak at 42 h. These findings suggest that the expression of Cry j 7 and Pru p 7 secretions in *P. pastoris* was effectively consistent with observations made in past reports.

**Figure 2:**
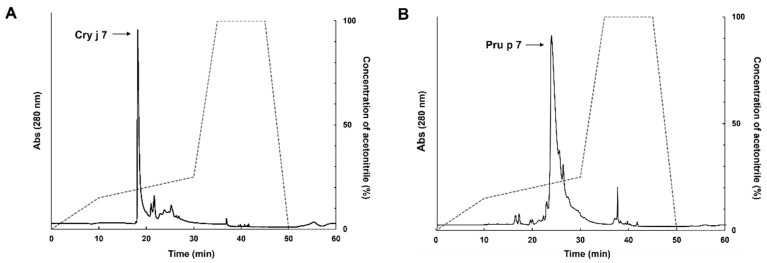
Purification of Cry j 7 (**A**) and Pru p 7 (**B**) by an RP-HPLC C18 column that was eluted with a linear gradient of 15–25% acetonitrile with 0.1% TFA. The black line shows the absorbance at 280 nm. The gray dotted line indicates the acetonitrile gradient.

At the end of the fermentation process, the supernatants of the culture were collected through centrifugation and applied to a cation exchange column as the first purification stage. The fractions containing the expressed proteins were further separated by using a PR-HPLC C18 column, and the purified products in each step were analyzed by tricine SDS-PAGE. Cry j 7 appeared as a single peak in the HPLC profile, with a retention time between 17.0 and 19.0 min (Figure 2A), confirming that it was isolated with high purity. By performing complete reduction, the hydrophobicity of these proteins increased, and they showed a slower elution time in RP-HPLC (Figure S2). In the case of Pru p 7, the main peak that was present from 23.0 to 27.0 min likewise showed good separation, even though other peaks occurred after 25.0 min (Figure 2B). The yield estimated from the absorbance at 280 nm for purified Cry j 7 was 20 mg from the 1 L fermentation cultivation medium, while it was 45 mg for Pru p 7 obtained from the 1 L culture. The purified samples were subsequently freeze-dried in liquid nitrogen and stored at −30 °C for further use in various experiments, including structural stability analysis and proteolysis research.

### 3.2. Characterization of GRP Proteins

**Figure 3:**
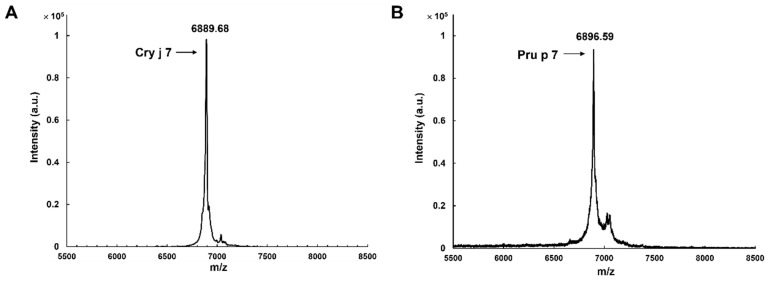
MALDI-TOF mass spectrum of the purified Cry j 7 (**A**) and Pru p 7 (**B**).

**Figure 4:**
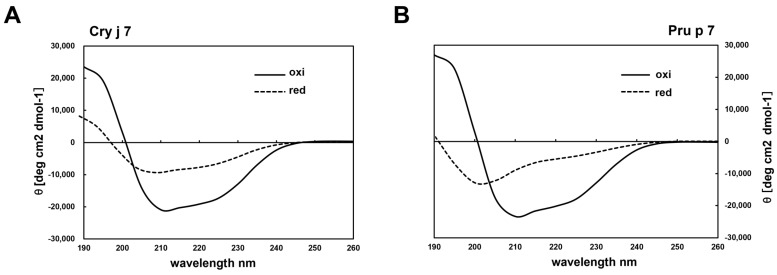
Circular dichroism spectra of Cry j 7 (**A**) and Pru p 7 (**B**). The proteins were dissolved in 50 mM PBS (pH 7.4) at 25 °C. Black solid line: oxidation state; black dotted line: reduction state.

Cry j 7 and Pru p 7, after being purified by RP-HPLC, were evaluated for their molecular weights via MALDI-TOF MS. The measured spectrum of Cry j 7 and Pru p 7 with six disulfide bonds (Figure 3) exhibited the desired peaks, corresponding to [M + H]^+^ at *m*/*z* 6889.68 and *m*/*z* 6896.59, respectively, which were within the expected error range. The far UV CD spectrum of Cry j 7 revealed a broad negative band with double peaks at approximately 208 and 222 nm, along with a minimum at 211 nm (Figure 4A). In contrast to the reduced state, the oxidized Cry j 7 displayed a primarily α-helical structure, whereas reduced Cry j 7 apparently transformed into a typical random-coil pattern, indicating a loss of its secondary structure. Pru p 7 demonstrated comparable CD findings as well (Figure 4B), indicating similar secondary structure content and folding of the oxidized and reduced states of proteins. Simultaneously, the ^1^H-NMR spectrum of Pru p 7 was also identical to the native one, which was extracted from peach pulp and purified by RP-HPLC (Figure S3). It is well known that the NMR chemical shifts of proteins are quite sensitive to tertiary structures [51]. The results strongly suggest that the disulfide bonds and three-dimensional structure of the purified GRP were identical to those of the natural GRP, consistent with the results of previous GRP studies.

### 3.3. Effects of pH and Temperature on the Secondary Structure of GRP Proteins

**Figure 5:**
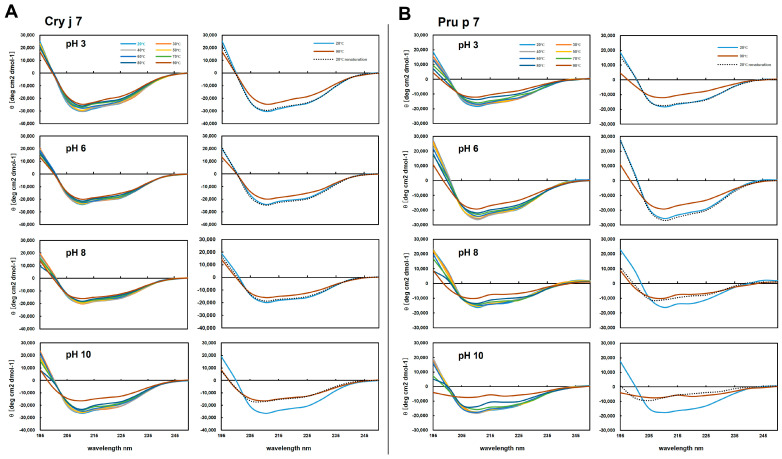
Secondary structural stability of Cry j 7 and Pru p 7 at different conditions, determined through circular dichroism. CD spectra of Cry j 7 (**A**) and Pru p 7 (**B**) heated in a stepwise manner in the range 20–90 °C and cooled back down to 20 °C at pH 3.0, pH 6.0, pH 8.0 and pH 10.0. The CD spectra at 20 °C, 30 °C, 40 °C, 50 °C, 60 °C, 70 °C, 80 °C, and 90 °C and after re-cooling to 20 °C (renaturation) are shown by light-blue, red, gray, orange, blue, green, dark-blue, dark-red, and black dotted lines, respectively.

The pH and thermal stability of Cry j 7 and Pru p 7 were examined by CD spectroscopy. At various pH values (3.0, 6.0, 8.0, and 10.0), both Cry j 7 and Pru p 7 formed structures rich in α-helical structures at 20 °C. However, as the temperature increased, GRP gradually underwent structural changes (Figure 5). Under acidic or neutral pH conditions, the pattern of the CD spectra did not change significantly even when the temperature increased, and when cooled to 20 °C, they re-turned to their original spectra. On the other hand, irreversible structural alterations were observed under basic pH conditions. In the case of Cry j 7 at pH 10.0, the profile of re-cooling at 20 °C displayed a similar result to that observed at 90 °C, maintaining the α-helix structure to a certain extent. For Pru p 7, the temperature values above 80 °C led to protein denaturation, and the CD spectrum showed an unfolded structure, which was not recovered after cooling down to 20 °C. These results show the differences in the stability of Cry j 7 and Pru p 7 again heating, and show that Pru p 7 is particularly unstable under alkaline conditions. Additionally, the calculated denaturation rate of the helical structure (Figure S4) suggested that Pru p 7 showed a greater degree of secondary structure transformation than Cry j 7 did at different pH levels. Overall, the secondary structure stability of Cry j 7 was higher than that of Pru p 7.

### 3.4. NMR Chemical Shift Assignment and Three-Dimensional Structural Analysis

^15^N-isotope-labeled Cry j 7 and Pru p 7 were expressed using the *P. pastoris* system in the BMM medium (Figure S5). The same purification procedure described in the Supplemental Materials and Methods was used to purify 1.5 mg of Cry j 7 and 3.5 mg of Pru p 7 from 1 L BMM medium. NMR spectroscopy provides extensive protein structural information [52]. The wide dispersion of the chemical shifts of amide protons in the ^1^H-^15^N HSQC spectra of oxidized Cry j 7 and Pru p 7 strongly suggested that these two proteins were correctly folded in the oxidized condition (Figure S6).

**Figure 6:**
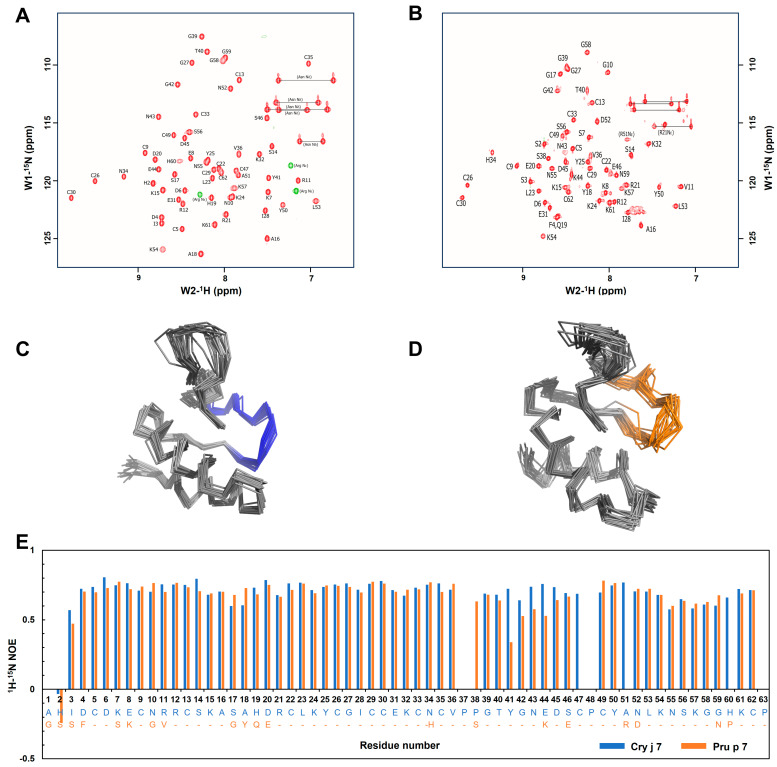
NMR investigation of Cry j 7 and Pru p 7. (**A**) ^1^H-^15^N HSQC NMR spectrum assignment of Cry j 7 in 10% D_2_O/H_2_O solution at pH 3.0, 25 °C. Asn Nε and Arg Nε are peaks from side chains. (**B**) ^1^H-^15^N HSQC NMR spectrum assignment of Pru p 7 in 10% D_2_O/H_2_O solution at pH 3.0, 25 °C. R21 Nε and R51 Nε are peaks from side chains. NMR structure of main chain of Cry j 7 (**C**) and Pru p 7 (**D**) in aqueous solution, and overlay of the ensemble of 20 final energy-minimized CYANA structures. The upper side is the C-terminus. (**C**) Gray: NMR structure of Cry j 7; blue: P38-C47 amino acid sequence. (**D**) Gray: NMR structure of Pru p 7; orange: the low-NOE region, i.e., the S38-C47 amino acid sequence. (**E**) Comparison of the heteronuclear steady-state ^1^H–^15^N nuclear Overhauser effect (NOE) values for Cry j 7 (blue) and Pru p 7 (orange).

To investigate the three-dimensional structure of Cry j 7 and Pru p 7 in the aqueous solution, chemical shift assignment was performed using the standard NMR experiments (Table S1). All backbone amide chemical shifts were assigned and are visually represented in the ^1^H-^15^N HSQC spectrum (Figure 6A,B). The set of 100 structures of Cry j 7 and Pru p 7 were calculated in the CYANA program, and the top 20 structures were extracted from the set based on the CYANA score function (Figure 6C,D). The structural calculation statistics are summarized in Table S2. The NMR structure and chemical shift information of Cry j 7 and Pru p 7 were deposited in the PDB with the codes 8X0R and 8X67, respectively. Both proteins formed well-converged structures rich in α-helices, in agreement with the CD data. The chemical shift values of the β-carbons confirmed that all cysteine residues formed disulfide bonds, which possibly contributed to the structural stability. Furthermore, the disulfide bond restriction, which was based on the X-ray crystallographic structure of another GRP, was evaluated in structural simulations to confirm the correct disulfide bond pairs between the 12 cysteine residues. The most energetically stable bond pairs were as follows: Cys5-Cys30, Cys9-Cys26, Cys13-Cys22, Cys29-Cys62, Cys33-Cys49 and Cys35-Cys47. The presence of a wide patch of positive charge in the C-terminal of the protein surface on the electrostatic potential surface can be observed (Figures S7A and S8A). The maps of molecular hydrophobic potentials on the surface of proteins (Figures S7B and S8B) indicate that Cry j 7 and Pru p 7, which were highly hydrophilic in the aqueous solution, play an important role in maintaining protein conformation.

The three-dimensional structures of Cry j 7 and Pru p 7 closely resemble each other, with a confirmed RMSD of 0.83 Å. The heteronuclear steady-state {^1^H}-^15^N NOE values revealed subtle differences between the helical segments of Cry j 7 and those of Pru p 7 (Figure 6E). A lower NOE value indicates greater flexibility in a particular region, and this was particularly evident in the P38/S38-C47 region, which is composed of α-helices and loops. In that specific region, Pru p 7 exhibited lower values, suggesting more mobility compared to Cry j 7, according to the results for it. Cry j 7 demonstrated a more converged structure in aqueous solutions at pH 3.0 and at room temperature, which was probably also related to this feature.

### 3.5. ^1^H NMR Analysis of Thermally Denatured GRP Structural Stability

**Figure 7:**
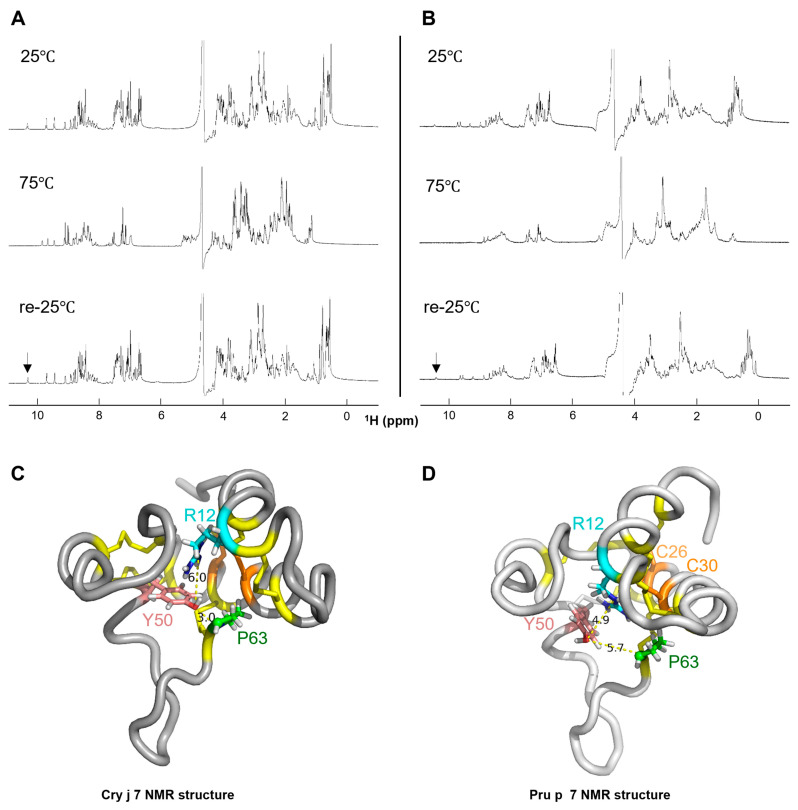
^1^H-NMR analysis of Cry j 7 and Pru p 7 in 10% D_2_O/H_2_O solution (pH 3.0) at various temperatures. The ^1^H NMR spectrum of Cry j 7 (**A**) and Pru p 7 (**B**) at 25 °C and 75 °C, and when re-cooled down to 25 °C. The arrow points to the peak of Tyr-OH. The atom distance (Å) between Y50 (pink color) and R12 (blue color)/P63 (green color) present in the NMR structure of Cry j 7 (**C**) and Pru p 7 (**D**). Gray: main chain. Yellow: disulfide linkage. Orange: C26 and C30.

The signal of one-dimensional NMR can reflect the folding state of proteins; thus, the structural stability of proteins can be evaluated. This enables a reliable distinction between various folding states of proteins and a discernment of subtle differences in structural stability [53]. Therefore, ^1^H-NMR was employed to assess the structural stability of Cry j 7 and Pru p 7 following thermal denaturation at an acidic pH level, pH 3.0 (Figure S9). As a result, the spectra of Cry j 7 and Pru p 7 exhibited distinct chemical shifts in the amide region at 25 °C and 75 °C, although the chemical shifts observed while cooling them down to 25 °C aligned well with the initial spectrum at 25 °C, signifying that Cry j 7 and Pru p 7 renatured to the same structural conformation as that before the thermal denaturation at 75 °C (Figure 7A,B). In the case of Cry j 7, the peak at a lower field than 10 ppm disappeared as the temperature reached 75 °C, and its absence in ^1^H-^15^N HSQC suggests that it is not an NH group. In our signal assignment, it was discovered that the side chain related to Y50 moved into the hydrophobic core of the structure (Figure 7C,D). This hydrophobic core protected the OH proton, resulting in a slower proton exchange rate, which allowed the OH signal to be easily detected in the ^1^H NMR spectrum. Besides the peaks around 9–10 ppm, the signals of C26 and C30 in the NH region also clearly changed. In particular, the peak signal intensities in Cry j 7 weakened, whereas the signal in Pru p 7 vanished. NOE signals were observed between R21 Hε and P63 Hγ for Y50 OH (Hη), suggesting that they are located close to each other. Therefore, the mobility of this region in Pru p 7 was likely increased under heating to 75 °C. It is also suggested that the structural stability of disulfide bonds between C26 (distance 3.0 Å) and C30 residues (distance 4.9 Å) close to the region was affected. As compared to Cry j 7, the conformation of Pru p 7 might have changed more as temperature increased, leading to a high exchange rate with water molecules, affecting signal detection in solutions. In general, the structural stability of Cry j 7 was higher than that of Pru p 7, as shown by thermal denaturation experiments at a low pH value.

### 3.6. Proteolytic Degradation of GRP Proteins

**Figure 8:**
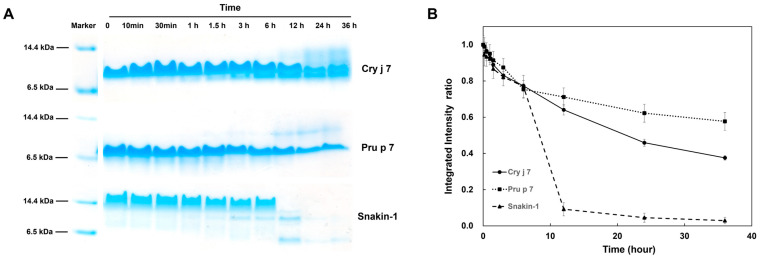
Kinetic of proteolysis of Cry j 7 and Pru p 7 (0.5 mg/mL) at 30 °C in the presence of 0.1 mg/mL thermolysin. (**A**) Representative analysis by tricine SDS PAGE gels. Lane mark: protein molecular weight mark. (**B**) Proteolysis kinetics. The data for Cry j 7, Pru p 7, and Snakin-1 are shown in circles, squares, and triangles, respectively.

Proteolysis is a valuable method with which to investigate protein dynamics and stability [54]. For this study, thermolysin, a common enzyme, was chosen to examine the proteolysis kinetics of Cry j 7 and Pru p 7 at 30 °C, and Snakin-1 (GRP from potato) was used as a control. In contrast to the result showing that Pru p 7 had one bond, the appearance of the second bond of Cry j 7 indicated that its native structure was digested into different fragments, and the primary band of Snakin-1 was the only one to vanish by the end of the experiment (Figure 8A). All three proteins exhibited slow degradation, showing a decrease in half-integrated intensity over several hours (Figure 8B).

The three proteins demonstrated different degrees of resistance to thermolysin, while the peptide Snakin-1 was shown to be the most sensitive, followed by Cry j 7 and Pru p 7. The resistance of GRP proteins to thermolysin revealed that Cry j 7 and Pru p 7 were almost completely resistant under these conditions, suggesting their high stability against proteolysis.

### 3.7. Stimulation of Gastrointestinal Digestion of GRP Proteins

**Figure 9:**
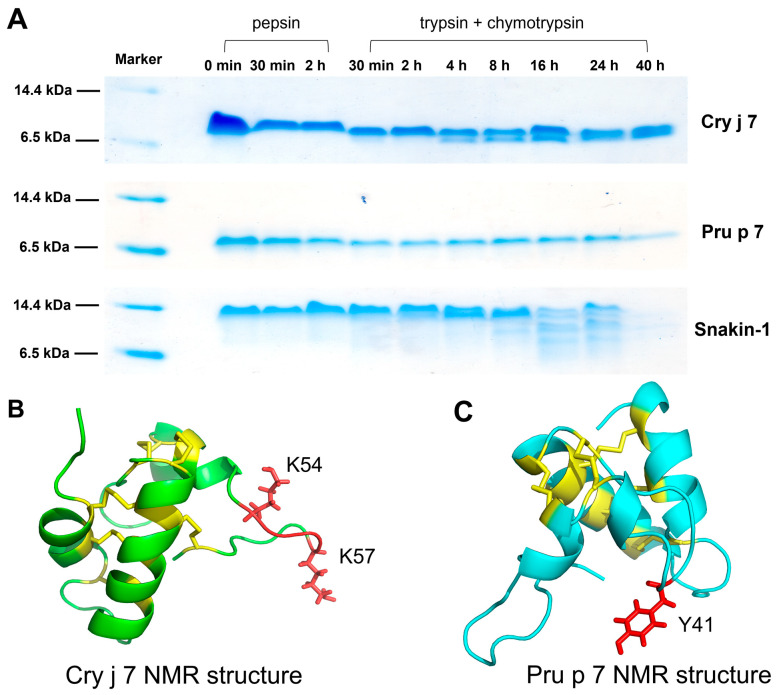
The simulated gastrointestinal digestion of Cry j 7 and Pru p 7. (**A**) Gastrointestinal digestion detected by tricine SDS PAGE. Pepsin digestion was performed in a pepsin-to-protein mass ratio of 1:20, and subsequent trypsin/chymotrypsin digestion was performed in a ratio of 1:50. Lane marker: protein molecular weight. Cleavage sites of trypsin/chymotrypsin digestion were presented in the NMR structure of Cry j 7 (**B**) and Pru p 7 (**C**). Green: Cry j 7 NMR structure. Blue: Pru p 7 NMR structure. Red: cleaved residues. Yellow: 6 disulfide bridges.

Food allergens generally have high structural stability, which allows them to retain an almost native structure and expose the surface epitopes even under environmental conditions capable of unfolding most globular proteins [45]. In our study, the peach GRP allergen Pru p 7 and the potato GRP peptide Snakin-1 demonstrated remarkable stability against gastric digestion by pepsin. Only one evident band was revealed by tricine SDS-PAGE, even after 2 h of digestion (Figure 9A). Subsequent treatment mimicking intestinal digestion resulted in effective Snakin-1 degradation, with the emergence of the digestion products and near-destruction after 40 h of reaction. In contrast, Pru p 7 was able to maintain an intact structure throughout the enzymatic degradation process, revealing that it could be highly stable in gastrointestinal digestion, with similar results to native Pru p 7 [46]. However, Cry j 7 is a pollen allergen that causes cross-reactivity with Pru p 7, sharing the same epitope regions. Therefore, Cry j 7 was also evaluated by gastrointestinal digestion for comparison. Following 4 h of trypsin and chymotrypsin enzyme digestion, a new band with a molecular weight of approximately 6.5 kDa appeared and remained consistent in the subsequent hours.

The results of the gastrointestinal digestion of Cry j 7 and Pru p 7 were also analyzed by RP-HPLC (Figure S10) and mass spectrometry (Figure S11). For Cry j 7, the peak in the chromatograms at 27 min, corresponding to the intact form, was detected during digestion by pepsin. After 4 h of trypsin and chymotrypsin digestion, an additional peak at 30 min corresponding to the proteolytic product was observed. Mass spectrometry analysis also revealed a peak at 27 min in all the samples, belonging to intact Cry j 7 stabilized by six disulfide bonds (*m*/*z* 6890.8). It also appears that one internal peptide bond was cleaved, i.e., the carboxyl end of K (15, 57 or 61), and a molecule (*m*/*z* 6906.4) with added water was produced. The other product, which was most likely the disulfide-stabilized protein without an amino acid sequence from K54 to K57 (Figure 9B), with a molecular weight of *m*/*z* 6451.9, was present at the peak at 30 min after 4 h of intestinal digestion (Figure S11A). For Pru p 7, a peak at 33 min was observed following intestinal digestion, and it was difficult to separate from the peak at 34 min, which was associated with gastric digestion and maintained the intact protein structure (*m*/*z* 6902.4). At the same time, a protein fragment with *m*/*z* 6917.5 was detected at the peak at 33 min (Figure S11B), implying that one internal amino acid in the loop area was digested, such as Y41 and K (54, 57 or 61). Y41 might be a cleavage site, given the reduction of the trypsin digestion fragment (Figure 9C).

GRP allergens exhibited sufficient structural stability in resisting degradation by gastrointestinal enzymes for a long time, which points to Pru p 7 as a food allergen that could induce immune responses through its conformational epitopes. In addition, Cry j 7 lost a short peptide (K54-K57) in its loop region and showed greater sensitivity to pepsin or trypsin and chymotrypsin digestion in contrast to Pru p 7.

### 3.8. Endosomal Degradation of GRP Proteins Is Mediated by Cathepsin S

**Figure 10:**
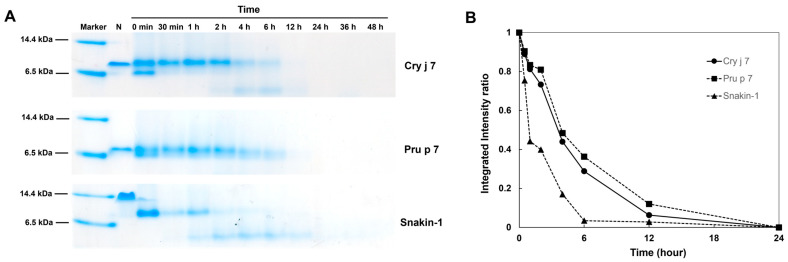
Degradation analysis of Cry j 7 and Pru p 7 in vitro. (**A**) Endosomal degradation assays by Cathepsin S enzyme in sodium acetate buffer with DTT; the degradation profile was analyzed by tricine SDS PAGE. Lane marker: protein molecular weight marker. Lane N: sample without DTT and Cathepsin S enzyme. (**B**) Proteolytic stability analysis: symbols are Cry j 7 (circles), Pru P 7 (squares), and Snakin-1 (triangle).

The capacity of potential allergens to withstand endosomal digestion can reflect the sensitization process, providing essential details about the structural stability of allergens [55]. Thus, to mimic endosomal degradation, the Cry j 7 and Pru p 7 proteins were subjected to digestion by Cathepsin, a lysosomal cysteine protease that acts on substrates with glutamine or lysine [56]. The proteins were fragmented into several pieces when incubated in a reaction buffer containing DTT at 37 °C (Figure 10). After 2 h of digestion, an obvious low-molecular-weight band appeared in Cry j 7, indicating that it was digested into further fragments, while Pru p 7 displayed lower degradation. All proteins were observed to be thoroughly digested after 12 h. Cry j 7 and Pru p 7 took a longer time for half-life degradation compared to Snakin-1, showing better stability. It was possible that they retained a small proportion of their ordered secondary structure to resist degradation under reduced conditions (Figure 3). In the absence of DTT, the proteins preserved a relatively intact band even after 48 h of degradation (Figure S12). These results reveal that the oxidized proteins exhibited a high ability to resist digestion, whereas the reduced proteins lost their stable structure, and were more susceptible to breakdown. In summary, the sensitization of Cry j 7 was higher than that of Pru p 7 in Cathepsin S enzyme digestion.

After RP-HPLC analysis (Figure S13), the majority of the degradation fragments of Cry j 7 and Pru p 7 were determined to correspond to the amino acid sequence regions I3/S3-R12, K24-V36, and L53-K61, based on the analysis of the MS data (Figure S14). The flexible loop regions of GRPs were inferred as the primary locations of Cathepsin S cleavage. Simultaneously, we used the ProPred web tool and IEDB to predict major MHC II binding regions of target allergens to identify T-cell epitopes. The most likely binding location in Cry j 7 and Pru p 7 among these epitopes was the sequence Y50-G58, which connected to HLA-DRB1*04:01 (Table S3). This obtained MHC II binding region may correspond to the sequence of degradation fragments, suggesting that relevant immune responses of the GRP proteins might be mediated by the epitope sequence of Y50-G58.

### 3.9. Epitope Prediction of Cry j 7 and Pru p 7

The binding of IgE antibodies to specific allergen epitopes is an essential stage in the onset of allergic responses. To assess the IgE epitope prediction, we examined publicly accessible epitope tools, selecting B-cell epitope prediction methods provided by IDEB. Based on amino acid sequences, Bepipred 3.0 predicted two linear epitopes regions: S14-R21, and N52/P52-K61 (Figure S15). In general, Bepipred is more effective at predicting nonspecific epitopes. Therefore, 3D structures of allergic proteins were used for predicting specific IgE epitopes with higher accuracy [47].

**Figure 11:**
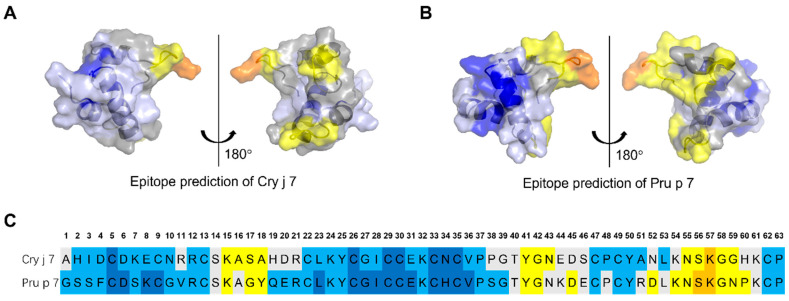
The epitope predictions for Cry j 7 and Pru p7. Three-dimensional structure of Cy j 7 (**A**) and Pru p 7 (**B**) inferred by the NMR experiment. At least three conformational epitope regions were predicted using the software DiscoTope 2.0. They are highlighted in yellow and orange. (**C**) Sequences of Cry j 7 and Pru p 7 with predicted AA involved in epitope regions. The color codes correspond to *DiscoTope* propensity scores. The higher the score, the higher the propensity to be an epitope. In following colors represent the values in descending order: blue, −20 or less; light blue, −20 to −15; gray, −15 to −12.5; yellow, −12.5 to −5.0; and orange, greater than −5.0.

Software DiscoTope 2.0 can be employed to predict the epitopic areas of Cry j 7 and Pru p 7. Despite the amino acid sequence being conserved, the predictions for these three potential epitope regions will differ due to their prediction based on the three-dimensional structure from NMR analysis (Figure 11). Three distinct epitopic regions were identified in Cry j 7 and Pru p 7. These regions were located as follows: the loop in the C-terminal region (around K54 to G59/N59, the upper right part of the molecule in the 3D model), and the loop region at the bottom of the molecule (Y41 to N43) and top part of the molecule (K15 to A18/Y18). Additionally, these three regions displayed greater flexibility in MD stimulation (Figure S16) and NOE results (Figure 6E) compared to other locations.

## 4. Discussion

Many organizations around the world are engaged in molecular allergology research, to acquire insights into the sensitization process of food and inhaled allergens. Following recent research, it has been shown that food allergies associated with pollen allergies, referred to as pollen-food allergy syndrome (PFAS), have become a public health concern as the prevalence has increased year by year. As the GRP allergens responsible for PFAS, the allergenicity and sensitization procedures for Cry j 7 and Pru p 7 are of great importance in investigations. Notably, we found that the allergic potential of GRP was not exclusively dictated by its quantitative content in the cypress family or the frequency of food consumption. Additional factors, encompassing allergen physicochemical properties, their biological activity, effects of structural stability and dynamics under stressful situations, as well as a patient’s individuality, play an important role in comprehensively exploring the mechanism of PFAS [11,17]. As research progresses, a more in-depth understanding of these factors should be developed to inform enhanced diagnostic and therapeutic strategies for PFAS.

In NMR research, the solution structures of Cry j 7 and Pru p 7 were determined. This was the first report of the NMR structural characterization of the Japanese cedar pollen allergen Cry j 7, and it also improved the intact three-dimensional structure of the peach allergen Pru p 7. Compared with the previously published three-dimensional structural model of Pru p 7 using a natural sample [46], we performed NMR experiments on stable isotope-labeled GRP proteins to obtain more accurate three-dimensional structure and flexibility information. In our experiments, all backbone resonances of Pru p 7 were completely assigned, and the presence of two brief helical stretches encompassing residues K44-E46 and P48-D52 were also identified in the C-terminal region. Furthermore, we determined the proper connection of six disulfide linkages among 12 cysteine residues. The disulfide bond between C13 and C22 was the same as that found in Pru p 7 NMR analysis, but C26 showed a tendency to form disulfide bond with C9 rather than C62 based on the CYANA calculation. Fortunately, the disulfide bridges involving C47, C30, C33, and C49 [46] were specifically distinguished: Cys5–Cys30, Cys33–Cys49, and Cys35–Cys47. Cry j 7 and Pru p 7 displayed high similarity in mainly helical structures in their N-terminal region but showed differences in the C-terminal flexible region according to the NMR results. In ^1^H-^15^N heteronuclear steady-state NOE, the significant difference in the P38/S38-C47 region revealed the high mobility of Pru p 7 and low mobility of Cry j 7, which corresponded to the molecular dynamic simulation results (Figure S16). In these flexible regions composed of loops and a short α-helical structure, there are differences in charge residues and hydrogen bonds, and these might affect the movement trajectories of atoms [57]. Furthermore, the impact of significant differences in the flexibility of this region on immunogenicity to allergens necessitates further exploration. These results are consistent with the results of thermal denaturation experiments using CD and ^1^H-NMR (Figure 5 and Figure S9), and clearly support the structural characteristics of Cry j 7, which is more stable than Pru p 7 in terms of physical chemistry. In particular, the finding that heating under alkaline conditions promotes the irreversible structural denaturation of Pru p 7 in contrast to what is observed for heating under acidic conditions is important when considering the removal of allergenicity through processing.

As is well known, stability in the digestive tract is an important factor related to the presentation of food allergen epitopes [58]. In contrast to pollen allergens, which are sensitized through the respiratory route, many food allergens are known to be more resistant to pepsin, a feature of a classical or true food allergen [59]. Interestingly, the degradation by enzymes, which is thought to occur in the digestive tract, showed the opposite trend to thermal denaturation, and Pru p 7 was more stable. Therefore, the difference in stability between these digestive enzymes may be mainly due to the specificity of the enzymes rather than structural stability. It is also worth noting that the results of the conformational epitope prediction for B cells include flexible loop regions that are susceptible to enzymatic cleavage (Figure 9 and Figure 11), although further IgE immunological experiments are still required to verify the true epitope region.

Sensitizations occur via the cutaneous or airway exposure of allergens, or when food allergens enter the human gastrointestinal tract to induce the immune response. In this study, we also evaluated the resistance to endosomal degradation associated with sensitization [55]. The characteristics of sensitization to allergens are strongly associated with endosomal digestion, the generation of allergen-derived peptides, and MHC II presentation [60]. Therefore, our investigation of the stability of Cry j 7 and Pru p 7 against endosomal degradation was expected to provide valuable insights into the sensitization in the GRP family. The observations in this work indicated that Cry j 7 and Pru p 7 were easily digested by Cathepsin S enzyme in the reduction state. This allowed the antigenic peptides to be effectively coupled with MHC II, inducing the Th2 response and promoting the production of specific IgE by B-cells [61]. However, the structure of Cry j 7 and Pru p 7 in the oxidation state remained hyperstable (Figure S12). The kinetics of proteolytic degradation were significantly influenced by the stability of antigens, which determined the density of appropriate antigenic peptide–MHC II complexes and, eventually, immunogenicity [62]. In certain instances, allergens with greater proteolysis resistance led to the lower production of T-cell epitopes, causing a decrease in immunogenicity [63]. Therefore, the efficiency of reduction of the GRP family after endocytosis may also be an important factor related to sensitization.

## 5. Conclusions

Analysis of three-dimensional structure and thermal stability using CD and NMR in this study revealed that Cry j 7 is more stable than Pru p 7. The differences in the mobility of the characteristic loop regions of the two proteins were clarified, and it was suggested that these loop regions not only contribute to stability but may also be epitope sites. In contrast, Pru p 7 had higher resistance to degradation by digestive enzymes, and analysis of the degradation fragments suggested that the difference in the primary sequence contributed to the resistance. In addition, although the resistance of endosomal enzymes involved in the establishment of sensitization was greatly reduced by the reduction in disulfide bonds in both proteins, the fact that the loop region in the native structure was cleaved to some extent suggests that the remaining structure after reduction may also have been related to the resistance. A detailed comparison of the structure and stability of the GRP family derived from pollen and fruit in this study will be useful as basic information for elucidating the mechanisms of sensitization and the development of allergies in PFAS in the future.

## Data Availability

The data presented in this study are available upon request from the corresponding author. The data are not publicly available due to privacy concerns.

## Supplementary Materials

The following supporting information can be downloaded at https://www.mdpi.com/article/10.3390/biom15020232/s1: Table S1. (A) NMR experimental details of Cry j 7; Table S1. (B) NMR experimental details of Pru p 7; Table S2. CYANA structure calculation statistics of Cry j 7 and Pru p 7; Table S3. (A) T-cell epitopes prediction of Cry j 7 allergen; Table S3. (B) T-cell epitopes prediction of Pru p 7 allergen; Figure S1. DNA sequences of recombinant plasmids; Figure S2. The RP-HPLC analysis of Cry j 7 (A) and Pru p 7 (B) under oxidation or reduction conditions; Figure S3. ^1^H-NMR analysis of the recombinant Pru p 7 and native Pru p 7; Figure S4. Circular dichroism spectra of Cry j 7 and Pru p 7 analysis at different pH values and temperature; Figure S5. The expression and purification of ^15^N-isotope-labeled Cry j 7 and Pru p 7; Figure S6. ^1^H-^15^N HSQC NMR spectra of Cry j 7 (A) and Pru p 7 (B) in 10% D_2_O/H_2_O solution, pH 3.0, 298 K. Figure S7. Protein structure characterization of Cry j 7; Figure S8. Protein structure characterization of Pru p 7; Figure S9. ^1^H NMR spectra of Cry j 7 (A) and Pru p 7 (B) at temperatures ranging from 10 °C to 70 °C in 10% D_2_O/H_2_O solution, pH 3.0; Figure S10. RP-HPLC analysis of stimulated gastrointestinal digestion of Cry j 7 and Pru p 7. Figure S11. MALDI-TOF MS spectra of the RP-HPLC fractions of gastrointestinal digestion of Cry j 7 (A) and Pru p 7 (B) for 4 h and 24 h; Figure S12. Degradation analysis of Cry j 7 and Pru p 7 in vitro; Figure S13. RP-HPLC analysis of Cathepsin S enzyme digestion of Cry j 7 and Pru p 7; Figure S14. MALDI-TOF MS spectra of the RP-HPLC fractions of Cathepsin S enzyme digestion; Figure S15. B-cell epitope prediction using the BepiPred-3.0 online software; Figure S16. Molecular dynamics simulation results of Cry j 7 and Pru p 7 at 300 k.
