# Supplementary material for: Structural and Stability Analysis of GRP Family Allergens Pru p 7 and Cry j 7, Which Cause Pollen and Food Allergy Syndrome
Source: Biomolecules. 2025 Feb 6;15(2):232. doi: 10.3390/biom15020232 (PMC11852976; doi:10.3390/biom15020232)
Supplement: Supplementary file 1 [file biomolecules-15-00232-s001.zip › biomolecules-3359718-supplementary.pdf]

Supporting information for

Structural Stability Analysis of GRP Family Allergens Pru p 7 and Cry j 7, Which Cause Pollen and Food Allergy Syndrome

Jingkang Zheng [1], Hiroyuki Kumeta [2], Yasuhiro Kumaki [2], Tomona Iizuka [1], Ichiho Yoshikawa [1], Ami Hanaoka [1], Tomoyasu Aizawa [1,*]

[1] Laboratory of Protein Science, Graduate School of Life Science, Hokkaido University, Sapporo 060-0810, Hokkaido, Japan.

[2] Faculty of Advanced Life Science, Hokkaido University, Sapporo 060-0810, Hokkaido, Japan

* Correspondence: aizawa@sci.hokudai.ac.jp (T. A.)

Materials and Methods

Construction of recombinant plasmid

A DNA fragment encoding Cry j 7 was amplified by PCR with a set of primers using synthetic pUC57-Kan vector as template. The Cry j 7 DNA sequence with primer, which includes the restriction enzyme recognition sequence utilized for introducing *Pichia. pastoris* and protease KEX2, a specific cleavage amino acid sequence in yeast. The primers were 5'-AAACTCGAGAAAAGAGCTCACATCGACTGCGACAAGGAGTGC-3' (forward primer), and 3'- ATGCTCGAGCCAAGCTCGGCGCGCATT-5' (reverse primer), with the *Xho I* and *EcoR I* site underlined, respectively. The PCR program was based on the instructions for KoD Fx Neo enzyme (TOYOBO, Japan) with a Tm of 68 °C and 35 cycles. The purified PCR product was digested with *Xho I* and *EcoR I* and ligated to the pPIC9 plasmid vector by using restriction sites. The ligated vector was transformed into *E. coil* DH5α competent cells. The method of pPIC9-Pru p 7 plasmid construction is same as pPIC9-Cry j 7. The primers of Pru p 7 were 5'-AAACTCGAGAAAAGAATTGSGGGAACACTTCAACAAAACGA-3' (forward primer), and 3'-GAATTCCTAAGGGCATTTGGGACCTCCTTCCTTA-5' (reverse primer), with *Xho I* and *Not I* enzyme site.

High-cell density fermentation of recombinant *P. pastoris*

High-cell density fermentation of *P. pastoris* was performed with a 5.0 L jar fermenter (MBF-500; Tokyo Rikaikikai, Japan), according to standard procedures. A single colony from a positive transformant was incubated in 5 mL of YPD medium overnight at 30 °C and 200 rpm. About 400 L of overnight cell culture was inoculated into 200 mL BMG medium (100 mM potassium phosphate pH 6.0, 1.34% YNB, 500×biotin, 1% glycerol) and cultured for 24 h at 30 °C, and then the resulting culture was inoculated into 2.4 L first medium (50% glycerol, 10×basal salt 840 mL, H_2O 1380 mL, PTM1 10 mL, 500×biotin 10 mL) in jar fermenter. During the fermentation, the temperature and agitation rate were maintained at 30 °C and 800 rpm, respectively; the pH was adjusted at 5.0 by adding 10% (v/v) ammonia water; the foam was decreased by an antifoaming agent (10% propylene glycerol). When the glycerol phase was completed, as identified by the sudden increase in the level of dissolved oxygen, then delivered second medium (50% glycerol 300 mL, PTM1 3.6 mL, 500×biotin 3.6 mL) at a speed of 30 mL/h to increase the cell mass. Within 7 h of the second medium feeding, a third medium (MeOH 900 mL, PTM1 10.8 mL, 500×biotin 10.8 mL) started at 3 mL/h rate. The feed was gradually increased until the maximum feed rate at 15 mL/h, and the whole cultivation time was about 42 hours. After putting methanol, 1 mL samples were collected every 12 h to confirm the cell density of target proteins. At the end of the fermentation process, the cells were centrifuged at 6000 rpm for 20 minutes, and the supernatants were frozen and stored at -30 °C.

Isotope-labeled Cry j 7 and Pru p 7 samples preparation

Baffled-flask cultivation was chosen as the culture method for isotope-labeled sample preparation. The ^{15}N isotope labeled reagent was used as the nitrogen source from CIL company (Cambridge Isotope Laboratories, Inc. USA). A single positive transformant colony from *P. pastoris*

expression system was grown at 30 °C about 28 h in a 300 mL shaking flask containing 50 mL BMD1 medium (100 mM potassium phosphate buffer, pH 6.0; 0.34%, w/v, YNB w/o amino acids and ammonium sulfate; 4 × 10^{-5}%, w/v, biotin; 1%, w/v, D-glucose and 1% w/v $^{15}NH_4Cl$) until OD_{600} = 2~6. The culture was centrifuged (3000 rpm, 5 min) and the cells were resuspended in the 100 mL growth medium BMD2 (100 mM potassium phosphate buffer, pH 6.0; 1.34%, YNB; 4 × 10^{-5}%, w/v, biotin, 0.5%, w/v, D-glucose and 0.2% w/v $^{15}NH_4Cl$). The growth phase was pursued for 12 h and stopped by centrifugation (3000 rpm, 5 min). Cells were washed with 0.2% glycerol solution immediately, collected by centrifugation before transferred into 500 mL induction medium BMM (100 mM potassium phosphate buffer, pH 6.0; 0.34%, w/v, YNB w/o amino acids and ammonium sulfate; 4 × 10^{-5}%, w/v, biotin, 1% w/v $^{15}NH_4Cl$ and 0.5% methanol). The culture was shaken at 30°C and 160-200 rpm. Methanol was added every 12 h to a final concentration of 0.5% (v/v) during the 72 hours induction period. The expressions of Cry j 7 and Pru p 7 were determined by tricine SDS-PAGE analysis. The ^{15}N-isotope labeled Cry j 7 and Pru p 7 samples were stored at -30°C after being purified according to the same protocols as these previously reported for non-labeled samples (2.1).

Sample preparation in the reduction state

To obtain the reduction state of Cry j 7 and Pru p 7, 1 M dithiothreitol (DTT) was added to 2–5 mg/mL Cry j 7oxi or Pru p 7oxi solution. The solution was then incubated at room temperature. After 16 h, the reaction solution was adjusted to pH 2.0~3.0 and purified by RP-HPLC through a linear gradient of 15–40% acetonitrile with 0.1% TFA, then lyophilized and stored at −30 °C before use. The procedure for getting the ^{15}N-isotope labeled reduced sample was described as above.

Native Pru p 7 sample preparation

The native Pru p 7 from peach pulp was extracted and purified according to the procedures published previously with some modifications. Frozen peal material was broken and dissolved in buffer and added equal volume of NaCl to final concentration 2 M. The mixture was filtered by different size filter membranes and collected the supernatants after centrifugation. The solution was diluted 40 times by Milli-Q and applied onto a Ni-NTA column (TOYOPEARL CM-650C) equilibrated with wash buffer (20 mM sodium phosphate buffer, pH 7.1). The target protein was eluted using elution buffer (20 mM sodium phosphate buffer, pH 6.3), and all fractions were collected after centrifugation at 5000 rpm, 1 minutes. Then the sample was filtrated, loaded onto a Cosmosil 5C18-AR300 column with a linear gradient 0–40% acetonitrile with 0.1% TFA for 80 minutes, and purified by RP-HPLC. After that, the freeze-dried native Pru p 7 sample were dissolved in a mixture of 10% D_2O solution with 50mM potassium phosphate buffer, pH 6.0 at 25°C to analyze the structure by ^1H-NMR.

^1H-NMR spectroscopy analysis of GRP proteins in thermal denaturation

Freeze-dried Cry j 7 and Pru p 7 samples were dissolved in a mixture of 10% D_2O/90% H_2O at pH 3.0. The temperature was incrementally adjusted from 10 °C to 75 °C using the different Chiller mode (0 °C ~ 20 °C: maximum; 20 °C ~ 30 °C: medium; 30 °C ~: off). NMR experiments were performed on a Bruker Avance III HD 600 MHz instrument.

Characterization of GRP proteins by mass spectrometry

Matrix-assisted laser desorption ionization time-of-flight mass spectrometry (MALDI-TOF) was carried out using a Bruker Autoflex Speed mass spectrometer (Bruker Daltonics). The protein samples were combined with sinapic acid matrix (α-Cyano-4-hydroxycinnamic acid in 0.1% TFA solvent) on a MALDI sample plate, and then the plate was loaded onto the spectrometer for analysis.

Molecular dynamics simulation

The molecular dynamics simulation (MD) of Cry j 7 and Pru p 7 was conducted in a water environment, using the TIP3 water model [1] . The system was prepared using CHARMM-GUI (https://www.charmm-gui.org/) with the appropriate protonation state representing pH 3.0 by calculating the pKa value of each residue using H++ website. The simulation was done using the CHARMM27 force field and computational package GROMACS 2023.2 version [2]. The three-dimensional NMR structures of Cry j 7 and Pru p 7 were used as the starting structure for the simulation. Sodium chloride ions with a concentration of 0.15 M were added to neutralize the system charge. All H-bonds were constrained using the LINCS algorithm. The electrostatic calculation was done using Particle-Mesh-Ewald (PME) grids, with a cut-off radius of 1.2 nm to reduce computing time. The system underwent an energy minimization using 50,000 steps of calculation [3]. After that, the system was equilibrated to 300 K for 100 ps in NVT ensemble, and the pressure to 1 bar for 100 ps using the NPT ensemble both using V-rescale thermostat and barostat respectively [4]. The equilibrated system was then subjected to 200 ns production simulation in NPT, 300K ensembles using V-rescale thermostat and Parinello-Rahman barostat and three replicas [5]. Trajectories and flexibility were evaluated through RMSD and RMSF of C using the trajectory from the last 50 ns.

Reference

[1] P. Mark, L. Nilsson, Structure and dynamics of the TIP3P, SPC, and SPC/E water models at 298 K, J. Phys. Chem. A. 105 (2001) 9954–9960. https://doi.org/10.1021/JP003020W.

[2] A.D. MacKerell, J. Nilesh Banavali, N. Foloppe, Development and Current Status of the CHARMM Force Field for Nucleic Acids, (2001). https://doi.org/10.1002/1097-0282.

[3] W.F. Porto, O.L. Franco, Theoretical structural insights into the snakin/GASA family, Peptides. 44 (2013) 163–167. https://doi.org/10.1016/J.PEPTIDES.2013.03.014.

[4] Q. Ke, X. Gong, S. Liao, C. Duan, L. Li, Effects of thermostats/barostats on physical properties of liquids by molecular dynamics simulations, J. Mol. Liq. 365 (2022) 120116. https://doi.org/10.1016/J.MOLLIQ.2022.120116.

[5] M. Kim, E. Kim, S. Lee, J.S. Kim, S. Lee, New method for constant- NPT molecular dynamics, J. Phys. Chem. A. 123 (2019) 1689–1699. https://doi.org/10.1021/ACS.JPCA.8B09082.

Table S1 (A). NMR experimental details of Cry j 7

NMR experiment	pulse program	scan number	f1 data size	f2 data size
[^{1}H-^{15}N] HSQC	hsqcetfpf3gpphwg	2	128	
[^{1}H-^{13}C] HSQC*	hsqcedetgpsp	16	512	
HNCO	hncogpwg3d	16	128	40
HNCA	hncagp3d	256	128	1
^{1}H-^{1}H NOESY**	noesyesgpph	8	1024	
^{1}H-^{1}H TOCSY**	mlevesgpph	8	1024	
^{15}N-NOESY	noesyhsqcf3gpwg3d	8	256	64
^{15}N-TOCSY	dipsihsqcf3gpsi3d	8	256	64

Table S1 (B). NMR experimental details of Pru p 7

NMR experiment	pulse program	scan number	f1 data size	f2 data size
[^{1}H-^{15}N] HSQC	hsqcetfpf3gpsi2	2	128	
[^{1}H-^{13}C] HSQC*	hsqcedetgpsp	16	512	
HNCO	hncogpwg3d	32	128	40
HNCA	hncagp3d	64	128	40
^{1}H-^{1}H NOESY**	noesyesgpph	8	1024	
^{1}H-^{1}H TOCSY**	mlevesgpph	8	1024	
^{15}N-NOESY	noesyhsqcf3gpwg3d	8	256	64
^{15}N-TOCSY	dipsihsqcf3gpsi3d	8	256	64

*The spectral width of the ^{13}C dimension was set at 80 ppm.

**With ^{15}N-decoipled.

Table S2. CYANA structure calculation statistics of Cry j 7 and Pru p 7

CYANA NOE assay	Cry j 7	Pru p 7
**Upper distance limits**		
Total	766	712
Short-range (|i-j| ≥ 1)	489	458
Medium-range (1 < |i-j| < 5)	201	198
Long-range (|i-j| ≥ 5)	76	56
**Dihedral angle limits**		
Total	91	81
Phi	46	40
Psi	45	41
**Average target function value**	0.02	0.05
**Violations**		
Distance > 0.1Å	0	0
Angle > 1˚	0	0
**RMSD (residue range 2..62)**		
Backbone atoms	0.63	0.8
Heavy atoms	0.98	1.46
**Ramachandran plot**		
most favored region	66.0%	76.9%
additionally allowed region	32.1%	17.3%
generously allowed region	1.95%	3.8%
disallowed region	0%	1.9%

Table S3 (A). T-cell epitopes prediction of Cry j 7 allergen

HLA type	Range	T-cell epitope	ProPred	IEDB
DRB1*01:01	41-49	YGNEDSCPC	+	+
	50-58	YANLKNSKG	-	+
DRB1*03:01	3-11	IDCDKECNR	-	+
DRB1*04:01	41-49	YGNEDSCPC	+	+
	50-58	YANLKNSKG	+	+
DRB1*07:01	50-58	YANLKNSKG	-	+
DRB1*08:01	23-31	LKYCGICCE	+	-
DRB1*11:01	50-58	YANLKNSKG	-	+
DRB1*13:01	23-31	LKYCGICCE	+	-
DRB1*15:01	50-58	YANLKNSKG	-	+
DRB5*01:01	50-58	YANLKNSKG	-	+
	53-61	LKNSKGGHK	+	+

Table S3 (B). T-cell epitopes prediction of Pru p 7 allergen

HLA type	Range	T-cell epitope	ProPred	IEDB
DRB1*01:01	25-32	YCGICCEKC	+	-
	50-58	YRDLKNSKG	+	+
DRB1*03:01	11-19	VRCSKAGYQ	-	+
	53-61	LKNSYGNPK	-	+
DRB1*04:01	25-32	YCGICCEKC	+	-
	50-58	YRDLKNSKG	+	+
DRB1*07:01	53-61	LKNSYGNPK	-	+
DRB1*08:01	41-49	YGNKEDCPC	+	-
DRB1*11:01	11-19	VRCSKAGYQ	+	-
	50-58	YRDLKNSKG	-	+
DRB1*13:01	23-31	LKYCGICCE	+	-
DRB1*15:01	50-58	YRDLKNSKG	-	+
DRB5*01:01	23-31	LKYCGICCE	+	-
	53-61	LKNSYGNPK	-	+

*Our analyses focused on the 9 common human alleles. The identified sequence length for the IEDB prediction method is 15, we extended the input sequence when its length was shorter than 15 residues.

A

GACTGGTTCCAATTGACAAGCTTTTGATTTAACGACTTTTAACGACAACTTGAGAAG
ATCAAAAAACAACTAATTATTCGAAGGATCCAAACGATGAGATTTCCTTCAATTTTTAC
TGCAGTTTTATTCGCAGCATCCTCCGCATTAGCTGCTCCAGTCAACACTACAACAGAAG
ATGAAACGGCACAAATTCCGGCTGAAGCTGTCATCGGTTACTCAGATTTAGAAGGGGA
TTTCGATGTTGCTGTTTTGCCATTTTCCAACAGCACAAATAACGGGTTATTGTTTATAAA
TACTACTATTGCCAGCATTGCTGCTAAAGAAGAAGGGGTATCTCTCGAGAAAAGAGCT
CACATCGACTGCGACAAGGAGTGCAACAGAAGATGCTCCAAGGCTTCCGCTCACGAC
AGATGCTTGAAGTACTGCGGTATCTGCTGCGAGAAGTGCAACTGTGTCCCACCCGGTA
CTTACGGTAACGAGGACTCTTGTCCATGCTACGCCAACTTGAAGAACTCCAAGGGTGG
TCACAAGTGTCCATAAGAATTCCCTAGGGCGGCCGCGAATTAATTCGCCTTAGACATGA
CTGTTCCTCAGTTCAAGTTGGGCACTTACGAGAAGACCGGTCTTGCTAGATTCTAATCA
A
GAGGATGTCAGAATGCCATTTGC

5’ AOX 1 primer site   α-factor   *Xho* I   KEX2 site   Cry j 7   *EcoR* I   3’AOX 1 primer site

B

GACTGGTTCCAATTGACAAGCTTTTGATTTAACGACTTTTAACGACAACTTGAGAAG
ATCAAAAAACAACTAATTATTCGAAGGATCCAAACGATGAGATTTCCTTCAATTTTTAC
TGCAGTTTTATTCGCAGCATCCTCCGCATTAGCTGCTCCAGTCAACACTACAACAGAAG
ATGAAACGGCACAAATTCCGGCTGAAGCTGTCATCGGTTACTCAGATTTAGAAGGGGA
TTTCGATGTTGCTGTTTTGCCATTTTCCAACAGCACAAATAACGGGTTATTGTTTATAAA
TACTACTATTGCCAGCATTGCTGCTAAAGAAGAAGGGGTATCTCTCGAGAAAAGAGGT
TCTAGCTTTTGCGATTCCAAATGTGGAGTCAGATGCAGTAAAGCTGGATACCAAGAAA
GGTGCTTGAAGTACTGTGGTATTTGCTGTGAGAAGTGTCATTGTGTTCCATCTGGTACT
TATGGGAACAAGGATGAATGTCCCTGTTATCGTGACCTTAAGAACTCAAAAGGCAATC
CTAAGTGTCCATAAGCGGCCGCGAATTAATTCGCCTTAGACATGACTGTTCCTCAGTTC
AAGTTGGGCACTTACGAGAAGACCGGTCTTGCTAGATTCTAATCAAGAGGATGTCAGA
AT
GCCATTTGC

5’ AOX 1 primer site   α-factor   *Xho* I   KEX2 site   Pru p 7   *Not* I   3’AOX 1 primer site

C

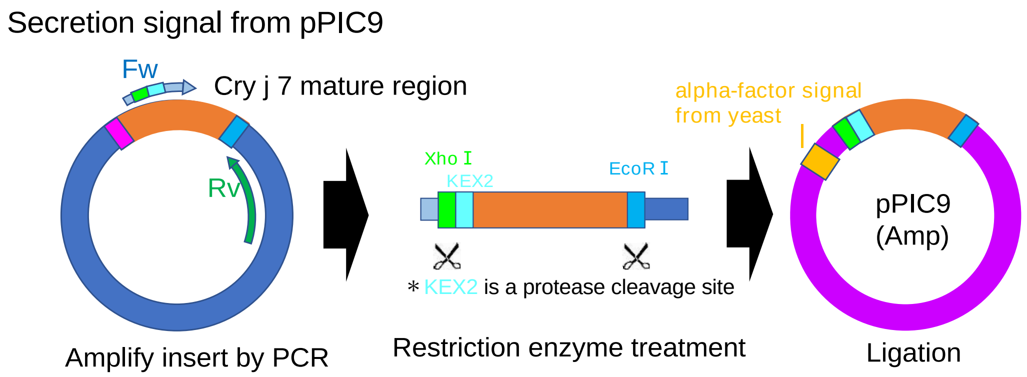

Figure S1. DNA sequences of recombinant plasmids. (A) pPIC9-Cry j 7. (B) pPIC9-Pru p 7.

(C) The vector construction of recombinant Cry j 7.

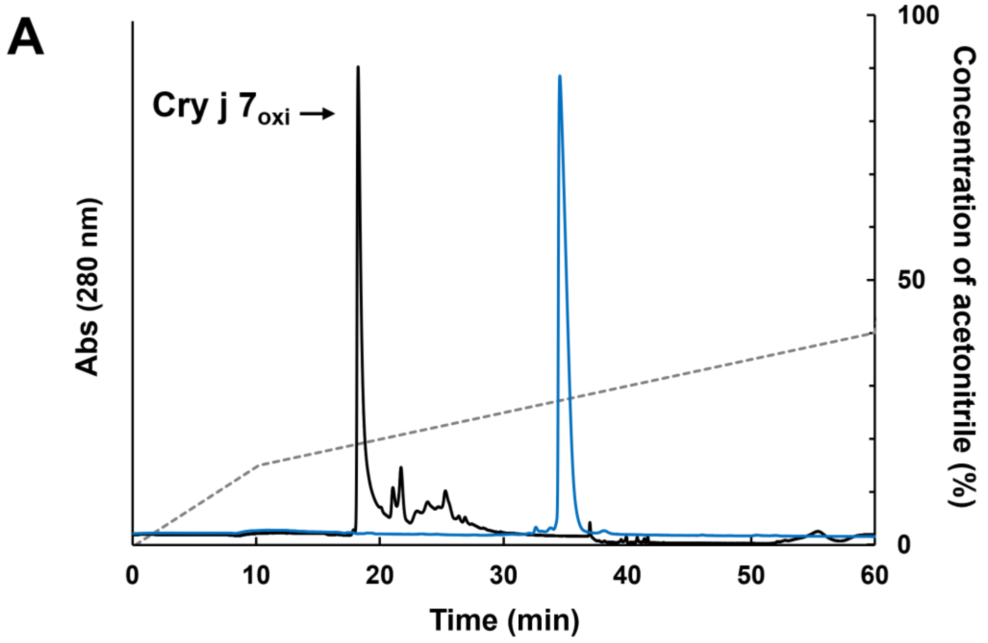

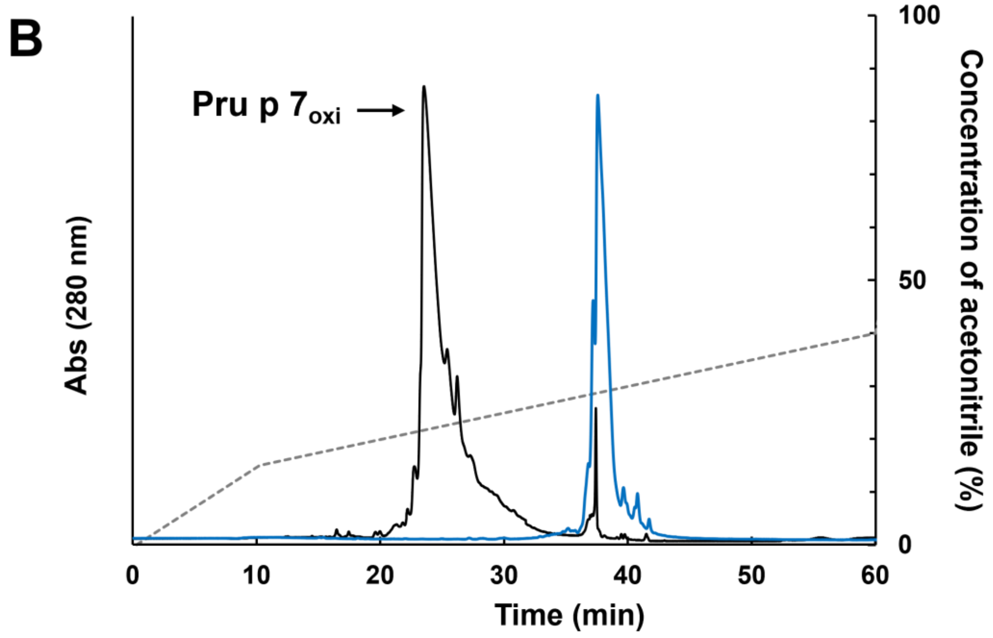

Figure S2. The RP-HPLC analysis of Cry j 7 (A) and Pru p 7 (B) under oxidation or reduction conditions. Purification of recombinant samples by a C18 column that was eluted with a linear gradient of 15-40% acetonitrile with 0.1% TFA. Black line: oxidation state. Blue line: reduction state.

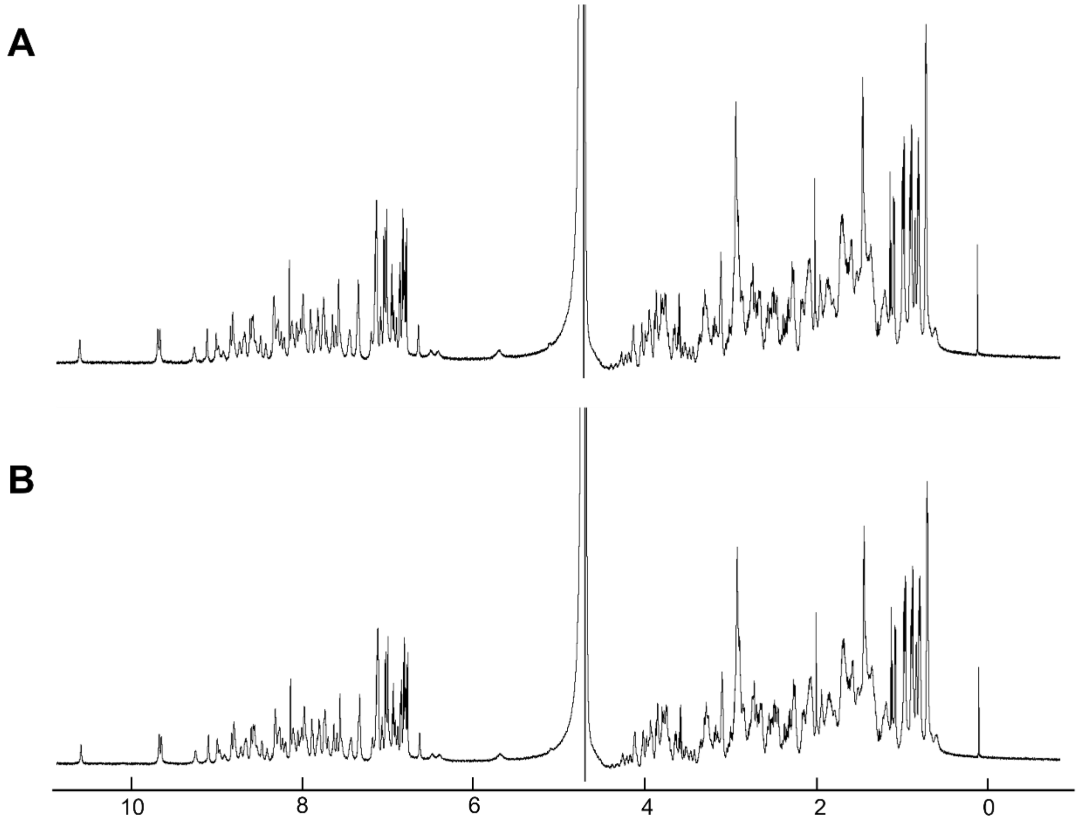

Figure S3. ^{1}H-NMR analysis of the recombinant Pru p 7 and native Pru p 7. The ^{1}H NMR spectra of recombinant Pru p 7 (A) and native Pru p 7 (B) in 50 mM potassium phosphate buffer, pH 6.0, with 10% D_2O, 298K.

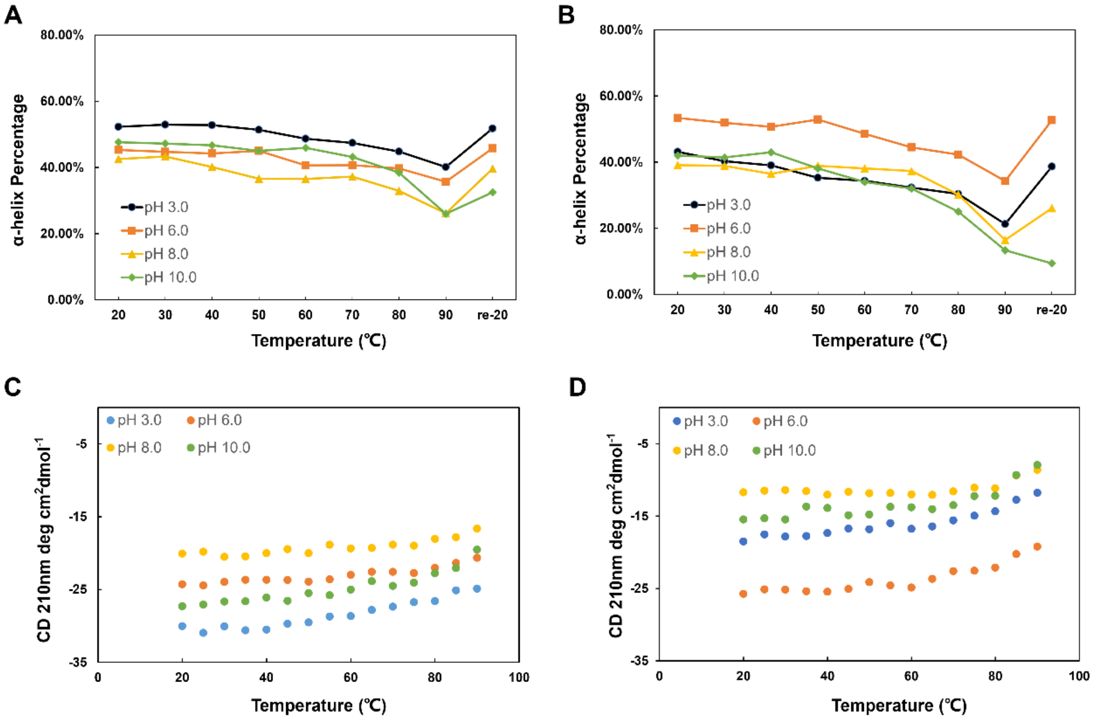

Figure S4. Circular dichroism spectra of Cry j 7 and Pru p 7 analysis at different pH values and temperature. The conformation analysis of Cry j 7 (A) and Pru p 7 (B) spectra through BeStSel website (https://bestsel.elte.hu/index.php). X-axis: the temperature ranges from 20 °C to 90 °C and back again to 20 °C. Y-axis: the α-helix structure proportion. The CD spectra values of Cry j 7 (C) and Pru p 7 (D) obtained by scanning at 210 nm for different temperature. Blue dotted: pH 3.0, orange dotted: pH 6.0, yellow dotted: pH 8.0, green dotted: pH 10.0.

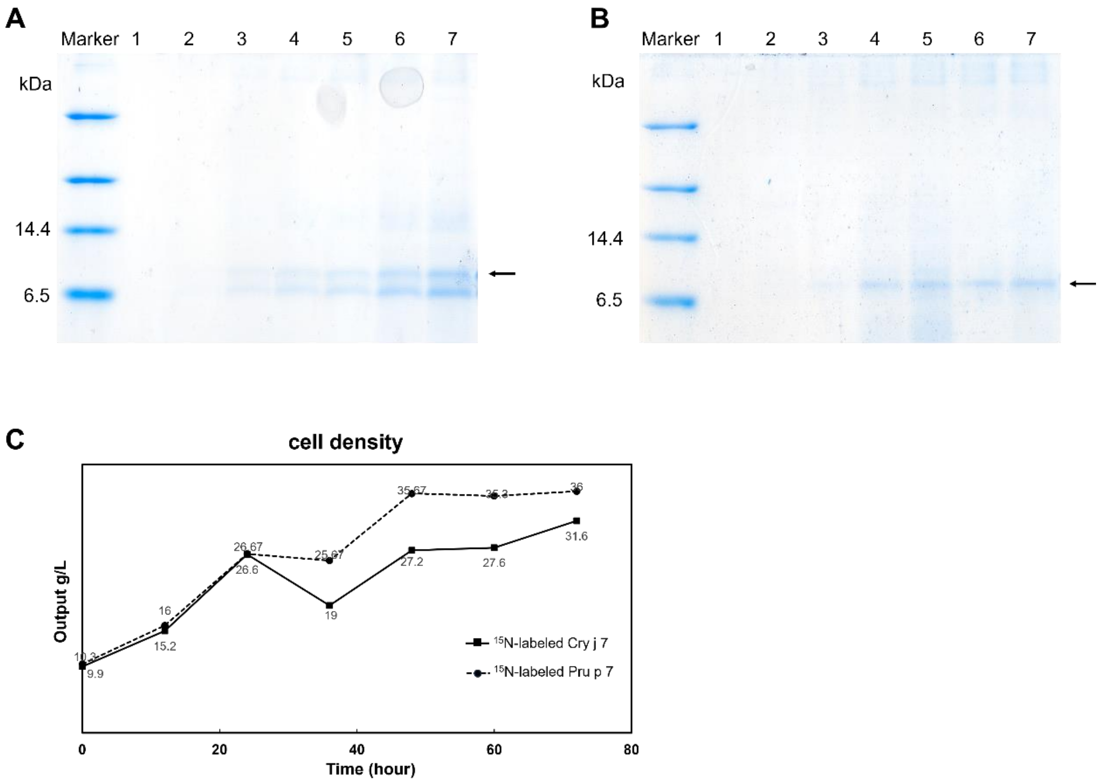

Figure S5. The expression and purification of ^{15}N-isotope labeled Cry j 7 and Prup7. Tricine SDS PAGE analysis of ^{15}N-isotope labeled Cry j 7 (A) and ^{15}N-isotope labeled Pru p 7 (B) in supernatants from *P. Pastoris* through baffled flask culture. Lane mark: protein molecular weight mark. Lanes 1-7: a total of 20 µL of supernatant samples taken at 0, 12, 24, 36, 48, 60, 72 hours of methanol-induction, respectively. (C) The cell density of recombinant protein in baffled flask cultivation for 72 hours.

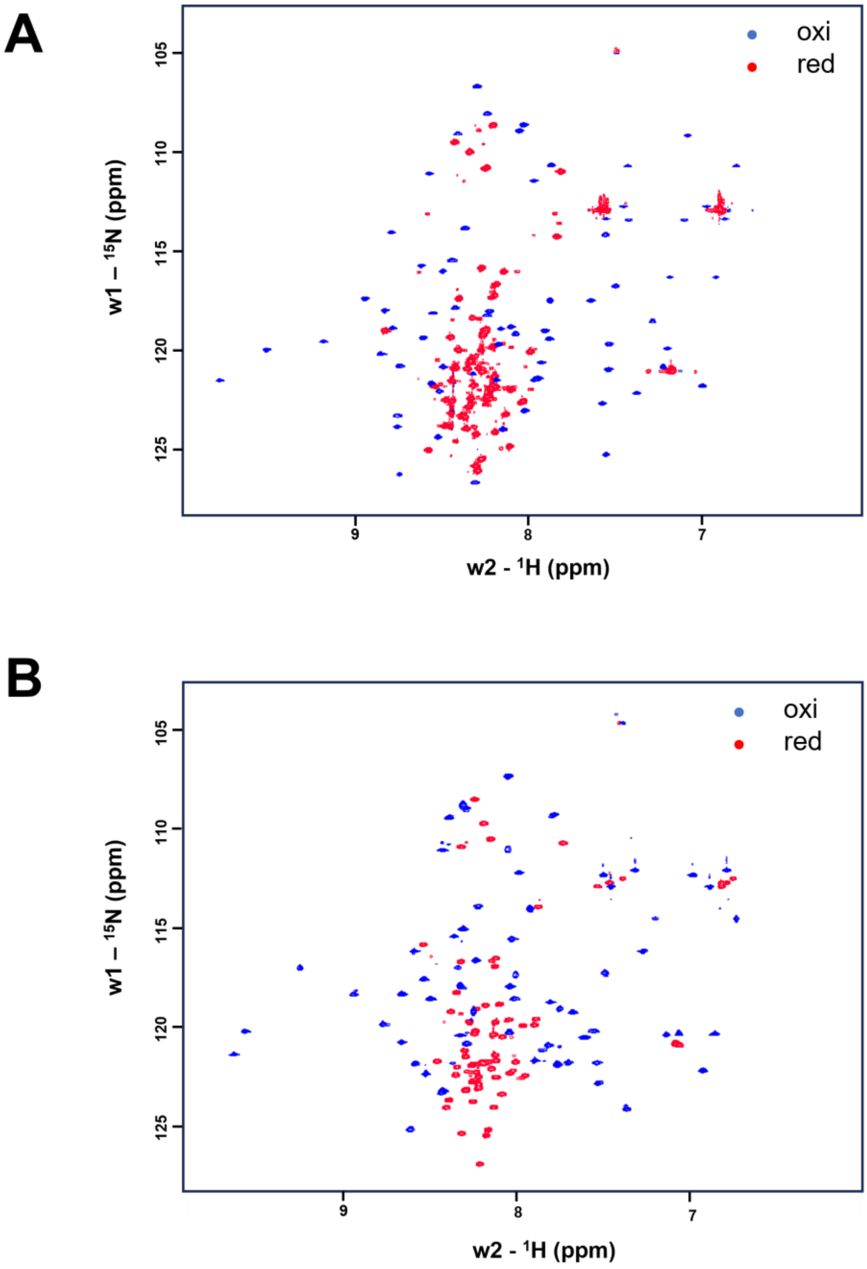

Figure S6. **^{1}H - ^{15}N HSQC NMR spectra of Cry j 7 (A) and Pru p 7 (B) in 10% D_2O/H_2O solution, pH 3.0, 298K. bule signal points: oxidation state; red signal points: reduction state.**

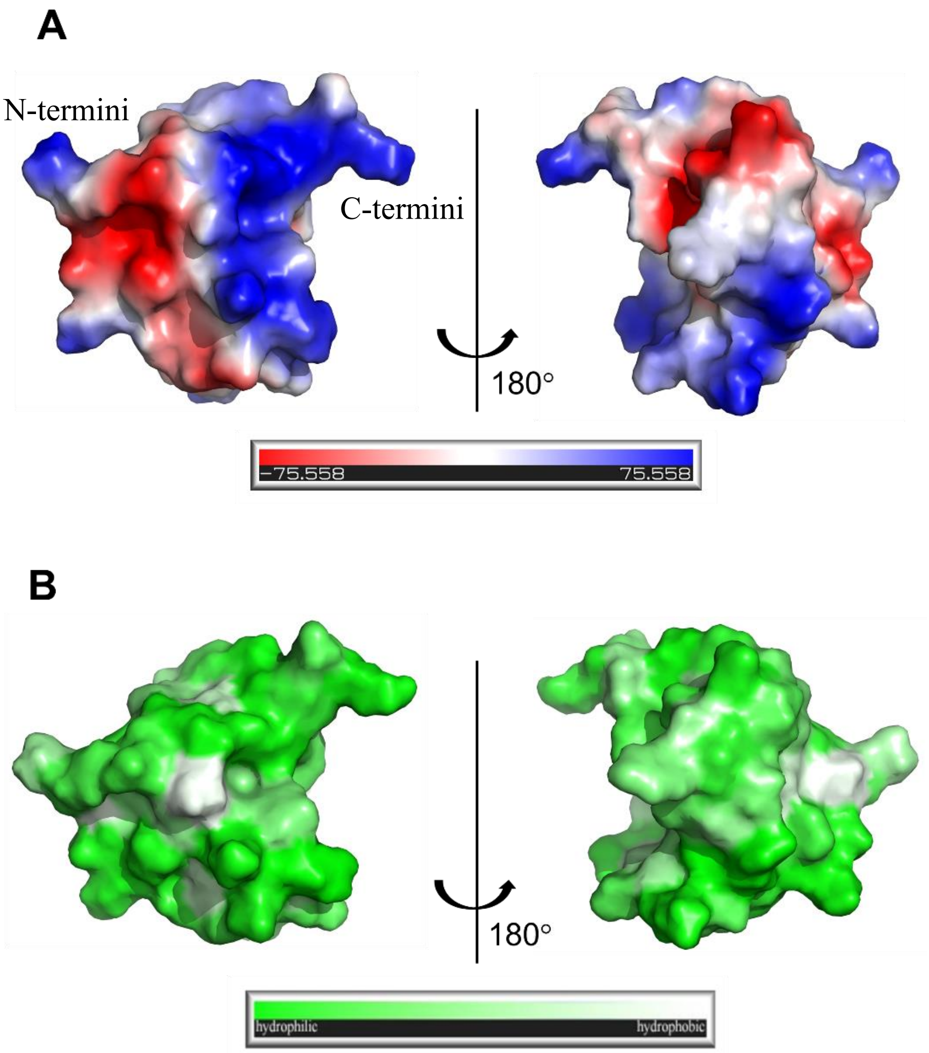

Figure S7. Protein structure characterization of Cry j 7. (A) Electrostatic potential at the surface of the Cry j 7. Hydrophilic and hydrophobic surfaces of the amphiphilic helix are represented. (B) Hydrophilic surface (green) and hydrophobic surface (white) of Cry j 7. This script colors the selection passed in based on the hydrophobicity scale as defined. *

***:** Eisenberg D, Schwarz E, Komarony M & Wall R (1984) Amino acid scale: Normalized consensus hydrophobicity scale. J. Mol. Biol.179:125-142.

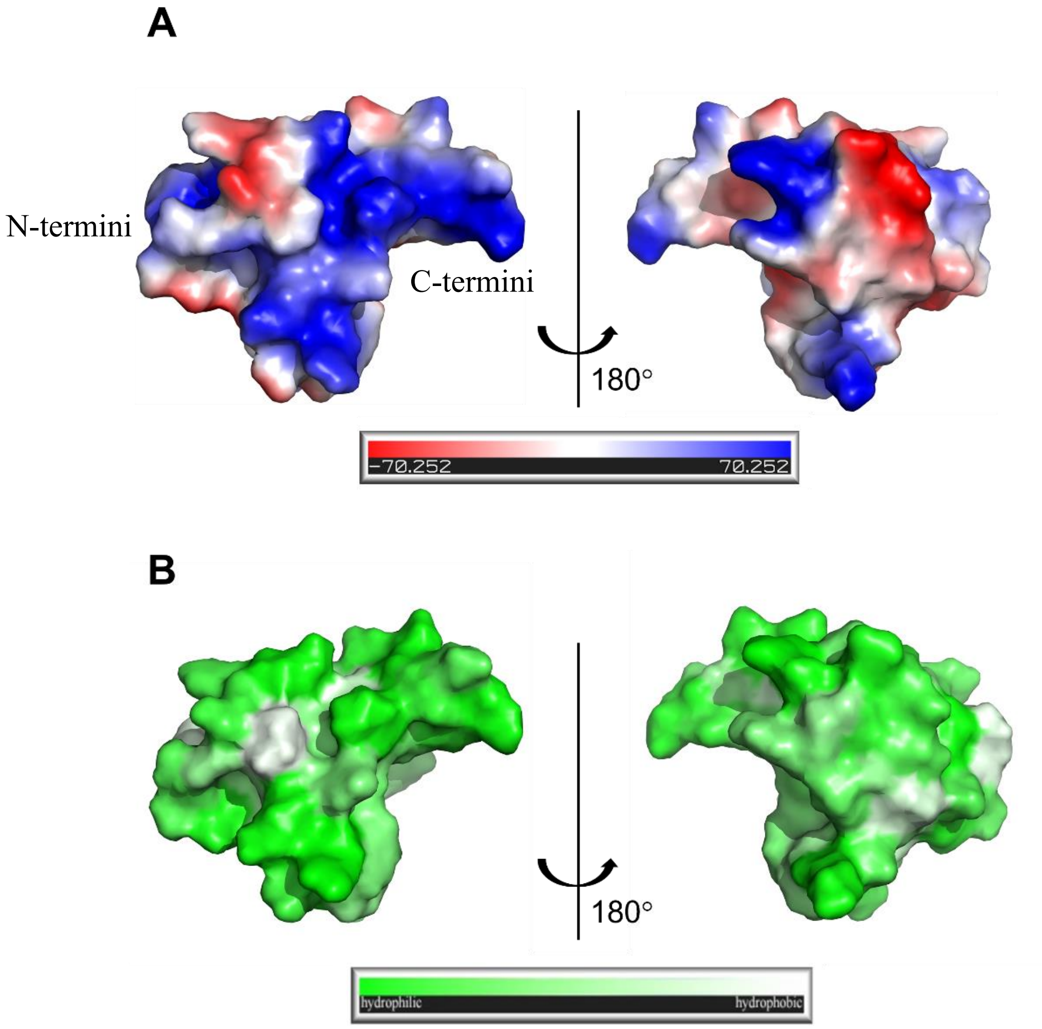

Figure S8. Protein structure characterization of Pru p 7. (A) Electrostatic potential at the surface of the Pru p 7. Hydrophilic and hydrophobic surfaces of the amphiphilic helix are represented. (B) Hydrophilic surface (green) and hydrophobic surface (white) of Pru p 7. This script colors the selection passed in based on the hydrophobicity scale as defined. *

***:** Eisenberg D, Schwarz E, Komarony M & Wall R (1984) Amino acid scale: Normalized consensus hydrophobicity scale. J. Mol. Biol.179:125-142.

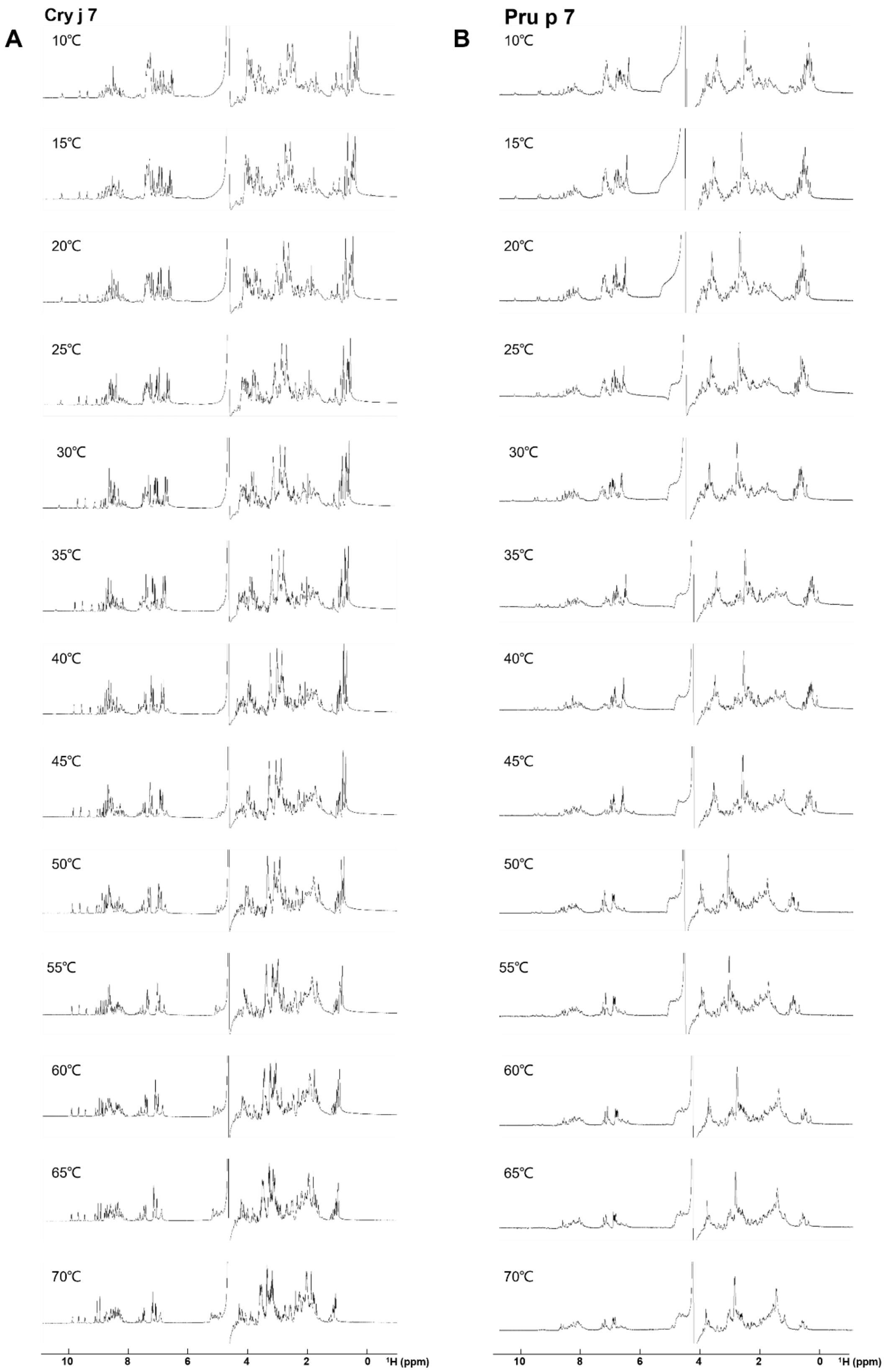

Figure S9. ^{1}H NMR spectra of Cry j 7 (A) and Pru p 7 (B) at temperatures ranging from 10 °C to 70 °C in 10% D_2O/H_2O solution, pH 3.0.

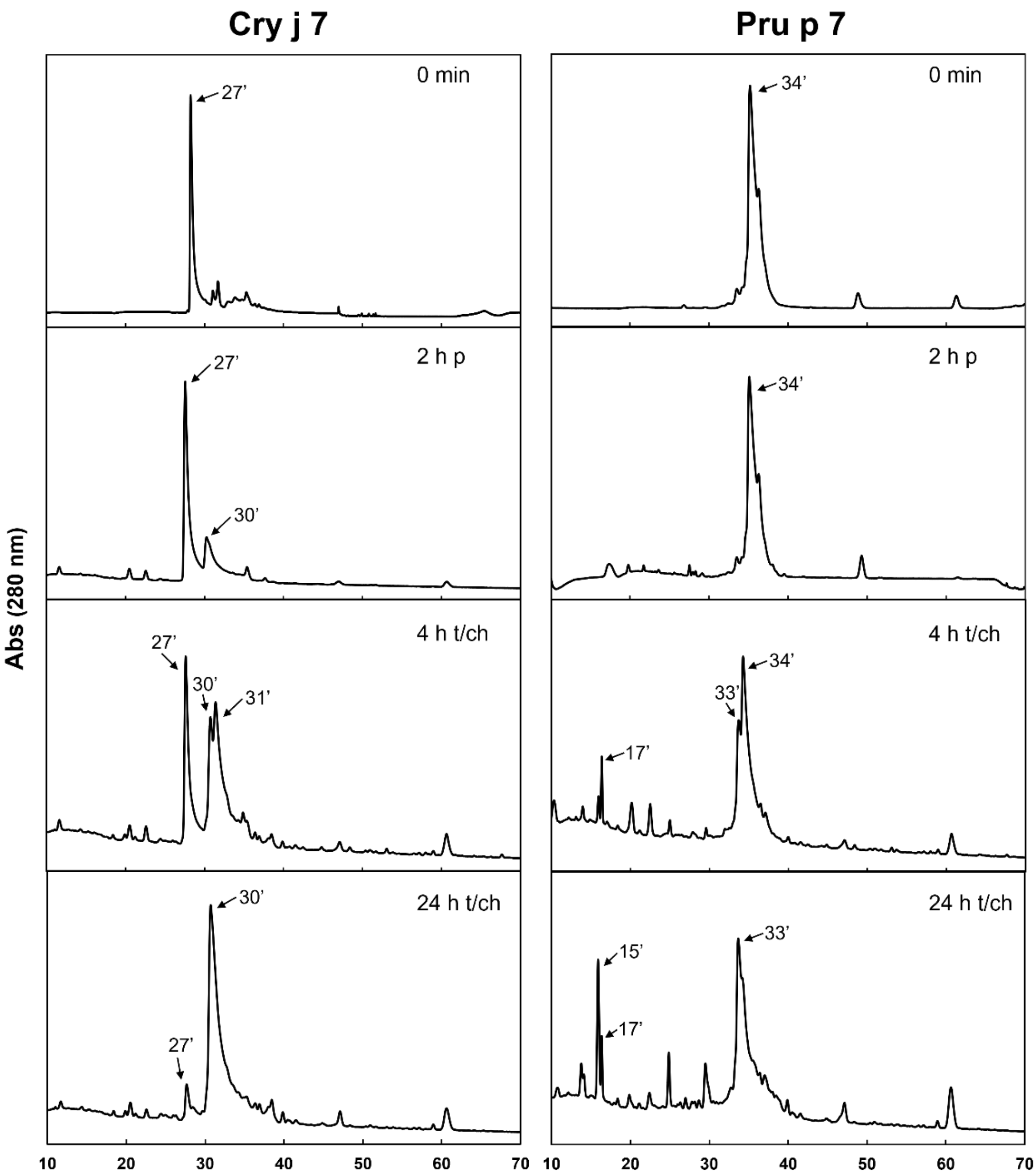

Figure S10. RP-HPLC analysis on stimulated gastrointestinal digestion of Cry j 7 and Pru p 7. RP-HPLC on C18 column with a linear gradient of acetonitrile concentration from 10% to 40% for 60 minutes (0min, 2 h p: pepsin digestion for 2 hours, 4 h t/ch and 24 h t/ch: subsequent trypsin/chymotrypsin digestion for 4 hours and 24 hours).

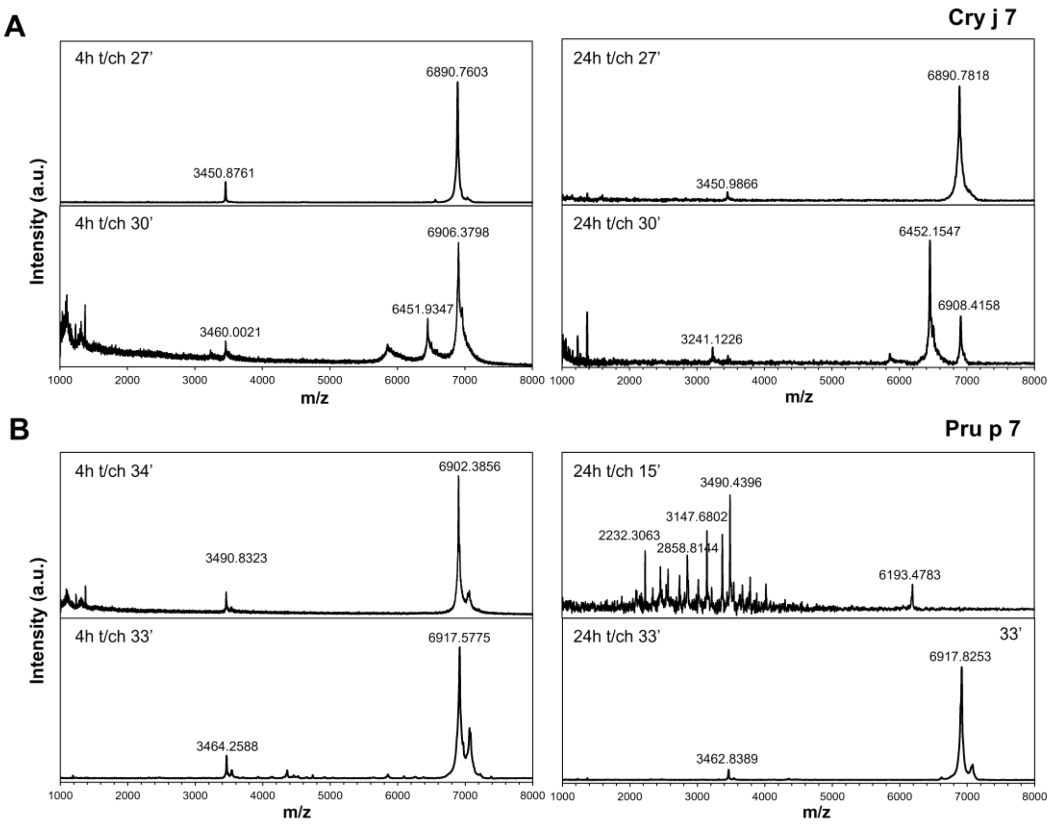

Figure S11. MALDI-TOF MS spectra of the RP-HPLC fractions of gastrointestinal digestion of Cry j 7 (A) and Pru p 7 (B) for 4 hours and 24 hours. The fractions of Cry j 7: 27 min and 30 min. Fractions of Pru p 7: 15 min / 34 min and 33 min.

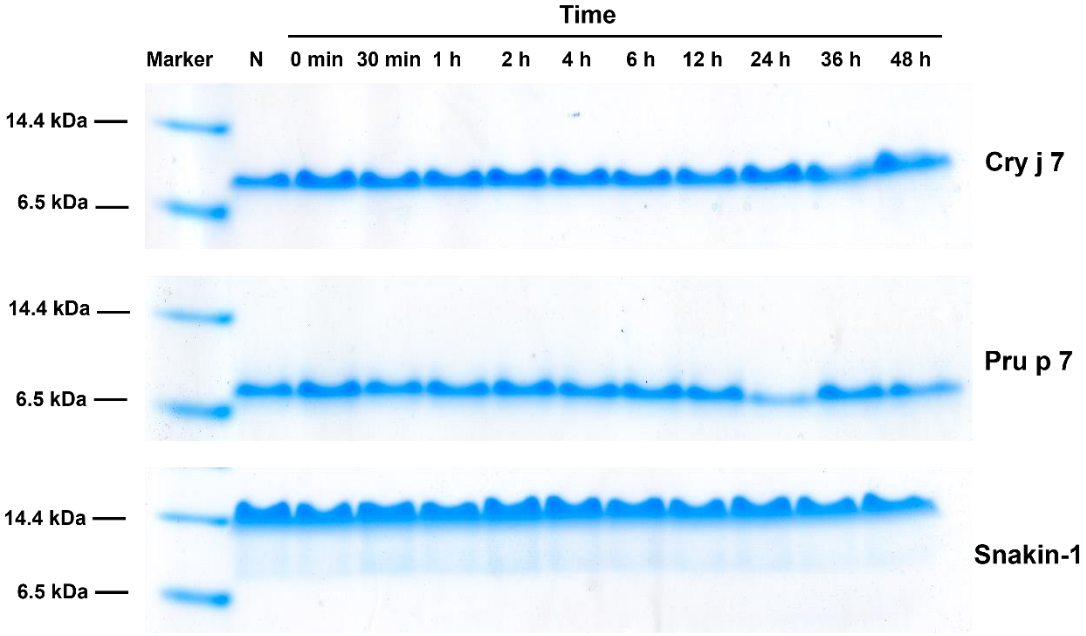

Figure S12. Degradation analysis of Cry j 7 and Pru p 7 *in vitro*. Endosomal degradation assay by Cathepsin S enzyme in sodium acetate buffer, pH 5.0 without DTT, and the degradation profile was analyzed by tricine SDS PAGE. Lane Marker: protein molecular weight marker. Lane N: sample without DTT and Cathepsin S enzyme.

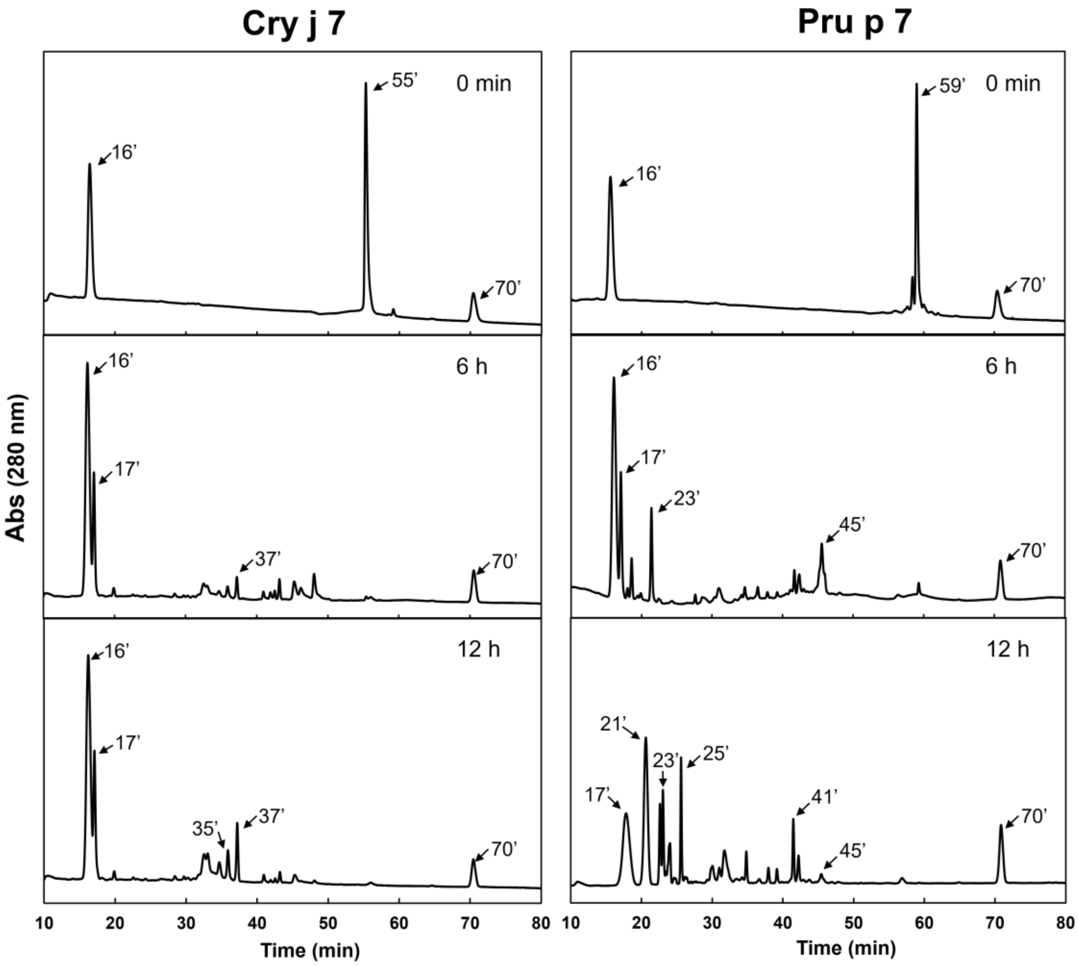

Figure S13. RP-HPLC analysis on Cathepsin S enzyme digestion of Cry j 7 and Pru p 7. RP-HPLC on C18 column with a linear gradient of acetonitrile concentration from 5% to 35% for 70 minutes (0 min, 6 hours, and 12 hours).

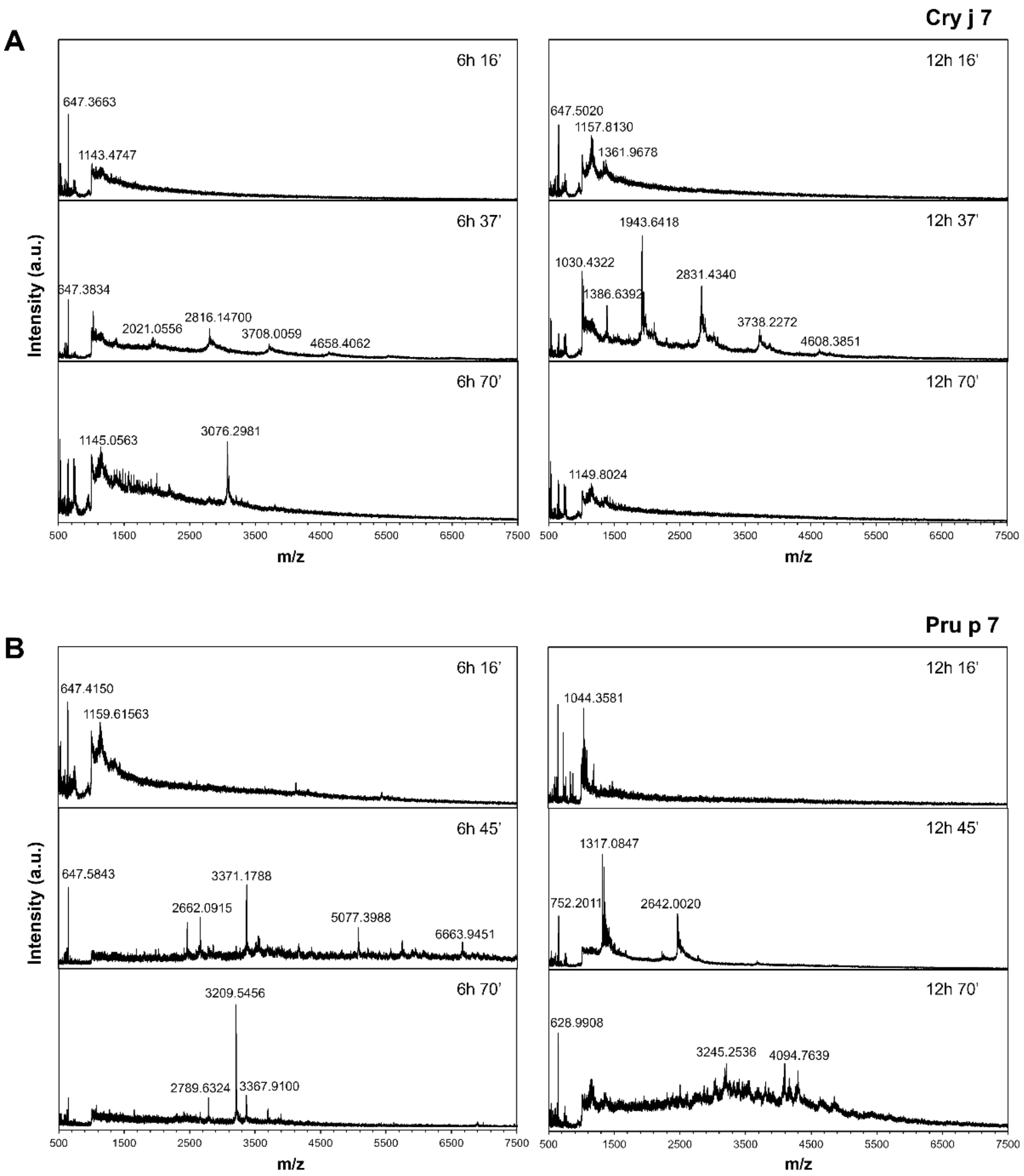

Figure S14. MALDI-TOF MS spectra of the RP-HPLC fractions of Cathepsin S enzyme digestion. (A) The HPLC fractions (16 min, 37 min, and 70 min) of Cry j 7 after Cathepsin S enzyme digestion for 6 hours or 12 hours. (B) The HPLC fractions (16 min, 45 min, and 70 min) of Pru p 7 after Cathepsin S enzyme digestion for 6 hours or 12 hours.

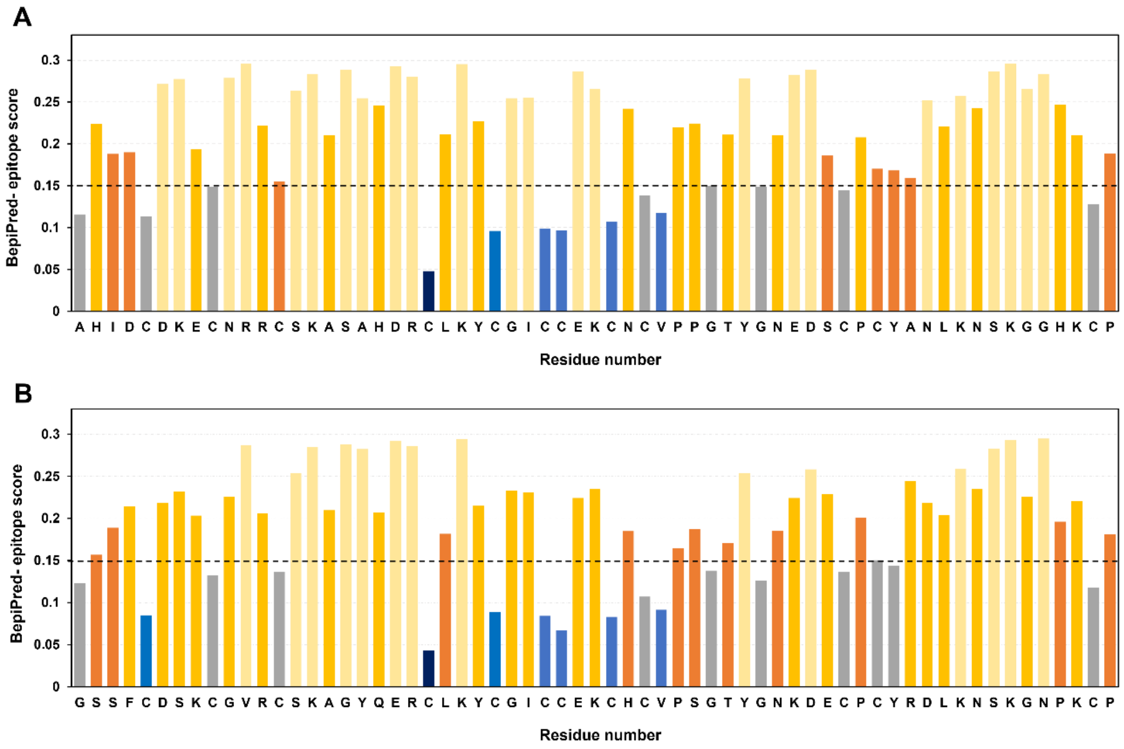

Figure S15. B-cell epitope prediction using the BepiPred-3.0 online software. (A) The B-cell epitope prediction graphic of pollen allergen Cry j 7. (B) The B-cell epitope prediction graphical of food allergen Pru p 7. In these diagrams, the x-axis and y-axis are protein sequence positions and BepiPred -3.0 epitope scores. Residues with higher scores are more likely to be part of a B-cell epitope. In ascending order: dark blue color (0-0.5), blue color (0.5-1.0), gray color (1.0-1.5), orange color (1.5-2.0), gold color (2.0-2.5), and light gold color (more than 2.5). The gray dotted line: threshold (0.15).

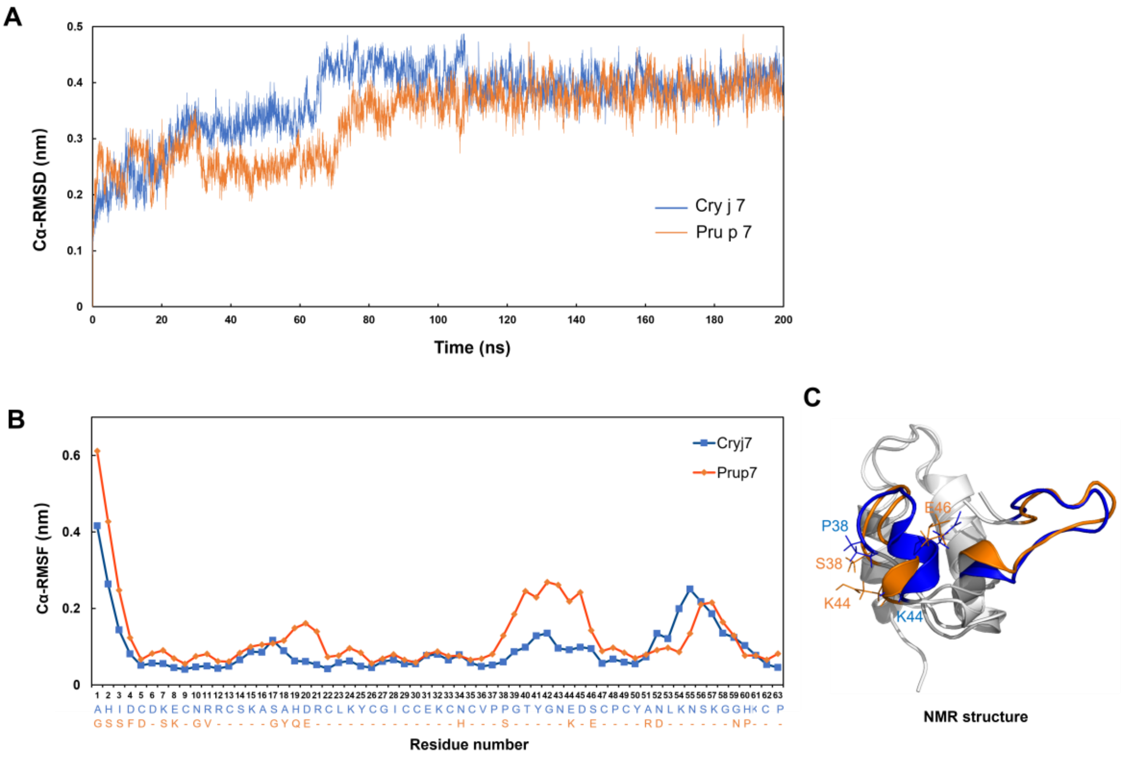

Figure S16. Molecular dynamics simulation results of Cry j 7 and Pru p 7 at 300 k. (A) RMSD plots for C-alpha after lsq fit to C-alpha of Cry j 7 and Pru p 7 throughout 200 ns simulation. (B) RMSF plots of Cry j 7 and Pru p 7 throughout the last 50 ns simulation. X-axis: amino acid sequence of Cry j 7 and Pru p 7; Y-axis: Cα-RMSF data. (C) NMR structural alignment of Cry j 7 and Pru p 7. Blue color: P38-S46, and A51 -K61 residues of Cry j 7. Orange color: S38-E46, and E51-K61 residues of Pru p 7. Blue color: Cry j 7. Red color: Pru p 7.
